# Inhibiting Type VI Secretion System Activity with a Biomimetic Peptide Designed To Target the Baseplate Wedge Complex

**DOI:** 10.1128/mBio.01348-21

**Published:** 2021-08-10

**Authors:** Y. Cherrak, I. Filella-Merce, V. Schmidt, D. Byrne, V. Sgoluppi, R. Chaiaheloudjou, S. Betzi, X. Morelli, M. Nilges, R. Pellarin, E. Durand

**Affiliations:** a Laboratoire d'Ingénierie des Systèmes Macromoléculaires, Institut de Microbiologie de la Méditerranée, UMR7255, Aix-Marseille Université—CNRS, Marseille, France; b Institut Pasteur, Structural Bioinformatics Unit, Department of Structural Biology and Chemistry, CNRS UMR 3528, C3BI USR 3756, Paris, France; c Protein Expression Facility, Institut de Microbiologie de la Méditerranée, FR3479, Aix-Marseille Université—CNRS, Marseille, France; d CRCM, CNRS, INSERM, Institut Paoli-Calmettes, Aix-Marseille University, Marseille, France; e Laboratoire d'Ingénierie des Systèmes Macromoléculaires, Institut de Microbiologie de la Méditerranée, UMR7255, INSERM, Marseille, France; f Sorbonne Université, Collège Doctoral, ED515—Complexité du Vivant, Paris, France; Pasteur Institute

**Keywords:** bacterial secretion system, type VI secretion system, T6SS, bioinformatic, biomimetic peptide, protein-protein interface, virulence inhibitor

## Abstract

Human health is threatened by bacterial infections that are increasingly resistant to multiple drugs. A recently emerged strategy consists of disarming pathogenic bacteria by targeting and blocking their virulence factors. The type VI secretion system (T6SS) is a widespread secretion nanomachine encoded and employed by pathogenic strains to establish their virulence process during host invasion. Given the conservation of T6SS in several human bacterial pathogens, the discovery of an effective broad-spectrum T6SS virulence blocker represents an attractive target for development of antivirulence therapies. Here, we identified and validated a protein-protein interaction interface, TssK-TssG, as a key factor in the assembly of the T6SS baseplate (BP) complex in the pathogen enteroaggregative Escherichia coli (EAEC). *In silico* and biochemical studies revealed that the determinants of the interface are broadly conserved among pathogenic species, suggesting a role for this interface as a target for T6SS inhibition. Based on the high-resolution structure of the TssKFGE wedge complex, we rationally designed a biomimetic cyclic peptide (BCP) that blocks the assembly of the EAEC BP complex and inhibits the function of T6SS in bacterial cultures. Our BCP is the first compound completely designed from prior structural knowledge with anti-T6SS activity that can be used as a model to target human pathogens.

## INTRODUCTION

During the past half-century, seven new antibiotic classes have been approved by the Food and Drug Administration (FDA) (1). None of them is efficient against the Gram-negative WHO priority list ESKAPE pathogens, namely, Enterococcus faecium, Staphylococcus aureus, Klebsiella pneumoniae, Acinetobacter baumannii, Pseudomonas aeruginosa and Enterobacter species, which are responsible for approximately 75% of infections and deaths by antibiotic-resistant bacteria (2, 3). In this postantibiotic era, new therapeutic options are required to fight against drug-resistant and life-threatening infections. The antivirulence strategy is a promising approach that seeks to disarm and neutralize pathogenic bacteria by interfering with bacterial virulence factors instead of growth pathways. Virulence factors are bacterial products that promote disease by either damaging the host or circumventing and evading the immune system (4). As they are not essential for growth, blocking virulence factors does not impose a strong evolutionary pressure on bacteria and hence could serve as an alternative or complement to traditional antibiotics (5–7). Virulence factors are numerous and include secretion apparatus involved in toxin translocation across the bacterial envelope (8). To date, nine secretion systems have been discovered, and the type VI secretion system (T6SS) is one of the most recently recognized examples (9–11). Genes encoding T6SS have been identified in more than 25% of sequenced Gram-negative bacteria, including pathogenic ESKAPE strains (12, 13). The T6SSs of P. aeruginosa, Aeromonas hydrophila, and Vibrio cholerae act on the host cytoskeleton to promote internalization (14) or to impair phagocytic functions (15, 16) and contribute to virulence in mouse models (17, 18). The T6SS promotes the intracellular spread of Francisella tularensis, Yersinia pseudotuberculosis, Burkholderia mallei, and Edwardsiella tarda, and T6SS mutants consistently exhibit a virulence defect *in vivo* (19–22). Similarly, the T6SS of Acinetobacter baumannii causes a host viability decrease in Galleria mellonella (23), while Salmonella enterica serovar Typhimurium, Shigella sonnei, and Vibrio cholerae employ the T6SS to disrupt the intestinal microbiota and colonize the host gastrointestinal tract (24–27). Although the T6SS is undoubtedly involved in pathogenesis initiation, strategies targeting this virulence factor are crucially lacking (28).

The T6SS belongs to the broad family of contractile injection systems, including bacteriophages, R-pyocins, and the metamorphosis-associated contractile structure (MAC) (29–32). The T6SS machinery includes a needle-like structure loaded with effectors and wrapped into a sheath built in an extended metastable conformation from an assembly platform, the baseplate (33). The T6SS contractile sheath is made of the TssB/C subunits surrounding an inner tube composed of HCP proteins and tipped by the VgrG puncturing spike (34–37). The cytoplasmic contractile tail is anchored to the bacterial cell wall through the TssJ-TssL-TssM transenvelope complex (38–40). Contraction of the tail leads to the perforation of the target cell and the delivery of a broad repository of effectors into both eukaryotic and prokaryotic cells (41–44).

The T6SS baseplate is a central piece of the T6SS machinery as it connects the tail to the membrane complex and initiates needle polymerization (33, 45–47). This complex includes the proteins TssF, TssG, and TssE, respectively, homologous to the T4 bacteriophage baseplate proteins gp6, gp7, and gp25, as well as TssK, which shares structural properties with siphophage receptor-binding proteins (36, 45, 46). TssK interacts with TssG, which is stabilized by TssF, resulting in the formation of the TssKFG wedge complex, representing an early T6SS baseplate building block homologous to the T4 baseplate wedge complex (45, 48–50). Recently, the high-resolution structure of the TssKFG complex from the pathogen enteroaggregative Escherichia coli (EAEC) has been solved and provided incisive insights into the structural organization of the protein complex (50, 51). This 500-kDa complex is composed of a monomeric TssG serving as a central backbone that interacts with a TssF dimer and two TssK trimers. While TssG contacts TssF proteins throughout its structure, the interaction with the two TssK trimers is localized and mediated by two small loops (foot 1 and foot 2) at the C terminus of TssG. Foot 1 (residues 216 to 252) and foot 2 (residues 300 to 330) form two triangular loops following the C3 symmetry of the TssK trimer and interacts with its N-terminal region (residues 1 to 18). Recently, we showed that overproduction of a truncated version of TssG lacking foot 1 and foot 2 interferes with baseplate assembly and leads to T6SS function impairment (50).

In this study, we deciphered the TssK-TssG interface of EAEC and assessed its relevance as a target to hinder T6SS-associated virulence. After validating the role of TssG feet in T6SS baseplate assembly and functioning, we investigated the sequence determinants of the TssK-TssG interacting region. Based on the available structures of the TssK-TssG interface and multiple-sequence alignments (MSA) of homologous proteins, we identified interacting motifs that we validated through site-directed mutagenesis coupled to functional assays. Guided by this structural and biochemical knowledge, we rationally designed a cyclic peptide able to interfere with T6SS baseplate biogenesis in EAEC. Furthermore, we carried out a comparative analysis of the predicted protein-protein interaction interface of TssK-TssG on 17 pathogens harboring at least one T6SS gene cluster. This analysis revealed a high conservation level of the TssK motifs targeted by our peptide as well as a preserved TssK-TssG interacting region that we further confirmed through cross-species protein pulldown experiments. Altogether, this study highlighted a conserved protein-protein interface that plays a key role in T6SS baseplate biogenesis and provided, to our knowledge, the first rationally designed T6SS inhibitor that could serve as a model to reinforce our arsenal against clinical pathogens and pave the way for new antivirulence inhibitors.

## RESULTS

### Validation of the TssK-TssG interface as a drug target.

**FIG 1:**
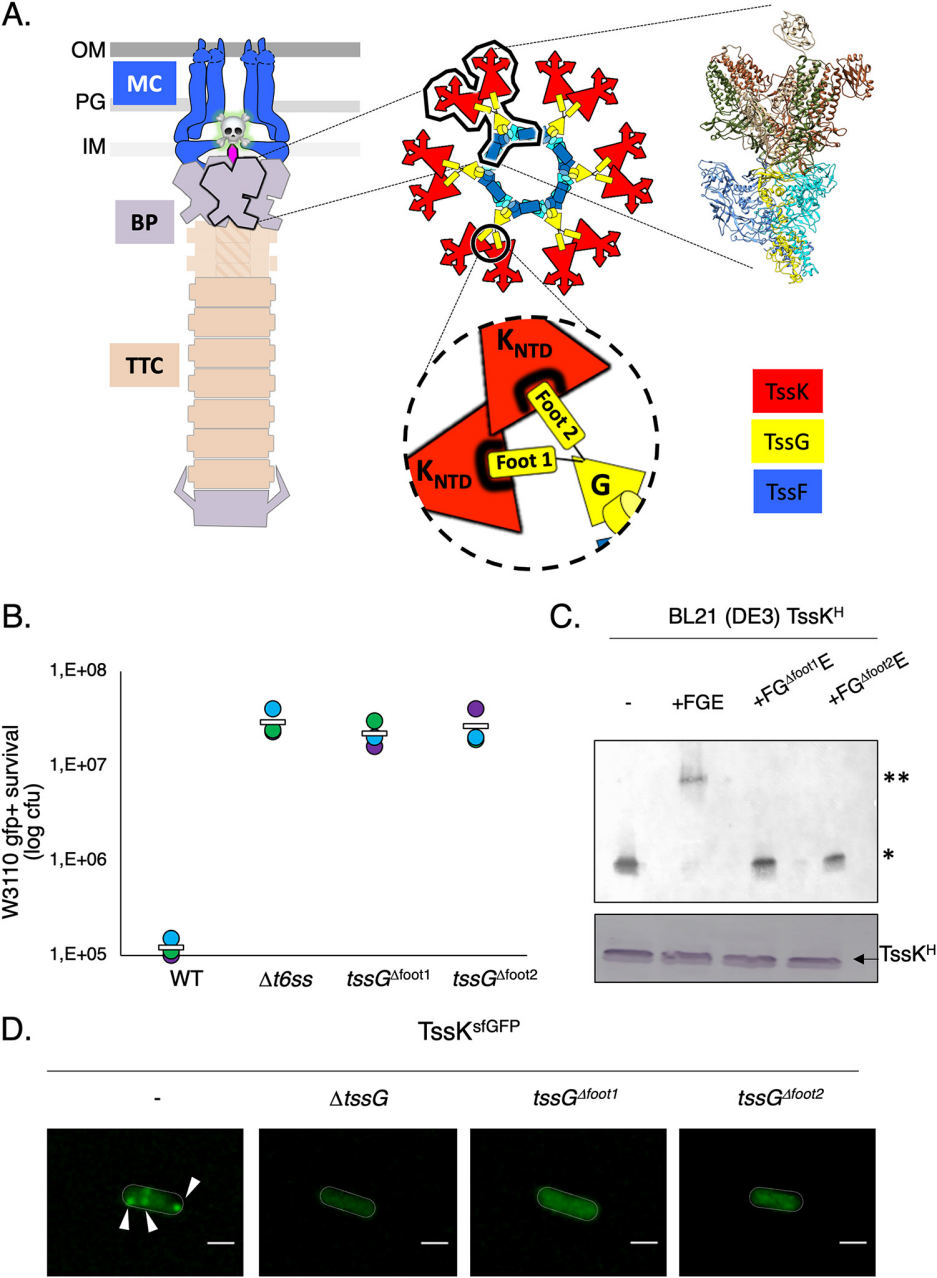
TssG foot domains are essential for T6SS functioning. (A) Architecture of the T6SS nanomachine. (Left) The different subcomplexes of the T6SS are presented: membrane complex (MC), baseplate complex (BP), and tail-tube complex (TTC). OM, outer membrane; PG, peptidoglycan; IM, inner membrane. (Center) Assembly of the BP, highlighting the three main components: TssK (red), TssG (yellow), and TssF (blue). The encircled inset shows the TssK-TssG interface. (Right) Structure of the fundamental unit, the wedge complex. (B) Antibacterial assay. The number of recovered Escherichia coli recipient cells (in log_10_ CFU), indicating their survival after 4 h of incubation against the indicated attacker cells. The assays were performed at least three independent times, with technical triplicates, and representative technical triplicate measurements (circles) are shown, with the corresponding average value calculated on all assays (white bar). (C) Native 4 to 16% gel analyzed by immunoblotting using anti-His antibodies on BL21(DE3) lysate expressing TssK-6×His, TssK-6×His plus TssFGE, TssK-6×His plus FG^ΔFoot1^E (*tssG* containing a deletion of the foot 1 domain), TssK-6×His plus FG^ΔFoot2^E (*tssG* containing a deletion of the foot 2 domain). The TssK-6×His plus TssFGE produced from BL21(DE3) cells shows the positions of two high-molecular-weight complexes (HMWCs) (*, TssK trimer; **, TssKFGE wedge complex). Formation of the HMWCs was monitored in a variant of TssG with either the foot 1 or foot 2 domain deleted. In each indicated construct, the TssK production level was analyzed by SDS-PAGE followed by immunoblotting and used as loading control (bottom). The native gel experiment was independently performed three times, and results of a representative experiment are shown. (D) Fluorescence microscopy recordings showing TssK-sfGFP localization in the absence of TssG, the TssG foot 1 domain (*tssG*^Δfoot1^), or the TssG foot 2 domain (*tssG*^Δfoot2^). The positions of foci corresponding to fully assembled baseplates are indicated by arrowheads. Microscopy analyses were independently performed three times, each in technical triplicate, and results of a representative experiment are shown. Bars, 1 μm.

We analyzed the effect of TssG foot mutation on T6SS biogenesis and functioning in the enteropathogenic organism EAEC. Using a chromosomally encoded and functional fusion protein of TssK and the superfolder green fluorescent protein (sfGFP), we monitored T6SS baseplate biogenesis by fluorescence microscopy in EAEC (45, 50). In this context, the deletion of both TssG feet abrogates the assembly of the baseplate (Fig. 1D and Fig. S1D). We proceeded with an in-frame chromosomal deletion of TssG foot 1 or foot 2, which resulted in the total loss of T6SS activity, highlighting the crucial role played by these two TssG structural elements (Fig. 1A and B). In order to diagnose at which stage the T6SS biogenesis was affected by TssG foot deletion, we biochemically analyzed the behavior of the TssKFGE unit using native gel experiments and observed that deletion of either foot 1 or foot 2 destabilizes the wedge complex (Fig. 1C and Fig. S1A and C). The TssG variants have no impact on the production and stability of TssF and TssK, which ruled out any indirect effect of TssG mutation on the stability of the wedge proteins (Fig. 1C and Fig. S1A and C). Based on the structure of the wedge complex, which displays a very localized interaction between TssG and TssK mediated by the feet, one could hypothesize that the deletion of the feet would uniquely impair the interaction between these two proteins. However, we found that the amount of TssG copurified using TssF-based pulldown was significantly lower (4.6% and 1.1% compared to 74.8%) when either of the 2 feet was deleted (Fig. S1B). We thus concluded that deletion of TssG foot 1 and foot 2 impairs the interaction of TssG with TssK and reduces the association with TssF. This was further confirmed using native polyacrylamide gel electrophoresis (PAGE) analysis, which showed that no complex intermediates were observable when foot 1 and/or 2 was deleted. Previous work suggested the importance of foot 1 and foot 2 in mediating the interaction with TssK through overproduction of TssG-derived interfering domains (50) or heterologous bacterial two-hybrid (BACTH) experiments (51). All this accumulated evidence indicates that any manipulations of TssG feet (i.e., deletion, mutations, and binding interference) has an important effect on the T6SS assembly and function and that the foot region of TssG represents a crucial target for therapeutic intervention that aims to block the T6SS assembly and functioning.

10.1128/mBio.01348-21.1 FIG S1 (A) Monitoring of TssF stability coproduced with TssG variants. Western blot analysis (SDS-PAGE) of E. coli BL21 cell soluble lysate overproducing the WT wedge complex (TssKFGE) and the mutated versions impaired in foot 1 (TssKFGΔFoot1E) or foot 2 (TssKFGΔFoot2E). The black arrow indicates the TssF-Strep protein upper band revealed by anti-Strep antibodies. The asterisk shows an unspecific cross-reacting band. (B) TssG variant pulldown by TssF. Soluble extracts of E. coli BL21(DE3) cells producing the TssKFGE wedge complex with TssGwt (left), TssGΔFoot1 (middle), or TssGΔFoot2 (right). TssF-Strep was used to pull down TssG variants using affinity purification. The level of TssG copurified with TssF is indicated below the panels. (C) TssK variant stability. Western blot analysis on BL21(DE3) lysate expressing TssK^S^, TssK^(W8A)S^, TssK^(L14A)S^, or TssK^(F19A)S^. The black arrow indicates the TssK^S^ variant protein revealed by anti-Strep antibodies. (D) Statistical analysis of TssK-sfGFP focus formation. Statistical analysis of TssK-sfGFP focus formation in various T6SS mutant backgrounds. Shown are box-and-whisker plots of the measured number of TssK-sfGFP foci per cell for each indicated strain, with the lower and upper boundaries of the boxes corresponding to the 25th and 75th percentiles, respectively (the center line shows the median value for each strain; whiskers extend 1.5 times the interquartile range from the 25th and 75th percentiles, and outliers are represented by dots. The number of cells analyzed for each strain is indicated on top. (E) Purification and biochemical characterization of the TssK variants. Analytical size exclusion chromatography analysis of the purified TssK protein and its variants on a Superose 6 column calibrated with 440-, 158-, and 75-kDa molecular mass markers (dotted lines). The molecular mass of each marker (in kilodaltons) is indicated on the top of the corresponding peak. The peak corresponding to the purified TssK protein (TssKS), the TssK variant mutated at position 8 [TssKS(W8A)], the TssK variant mutated at position 14 [TssKS(L14A)] and the TssK variant mutated at position 19 [TssKS(F19A)] are in blue, red, and green, respectively. Download FIG S1, TIF file, 1.7 MB. Copyright © 2021 Cherrak et al. 2021 Cherrak et al. https://creativecommons.org/licenses/by/4.0/ This content is distributed under the terms of the Creative Commons Attribution 4.0 International license.

**FIG 2:**
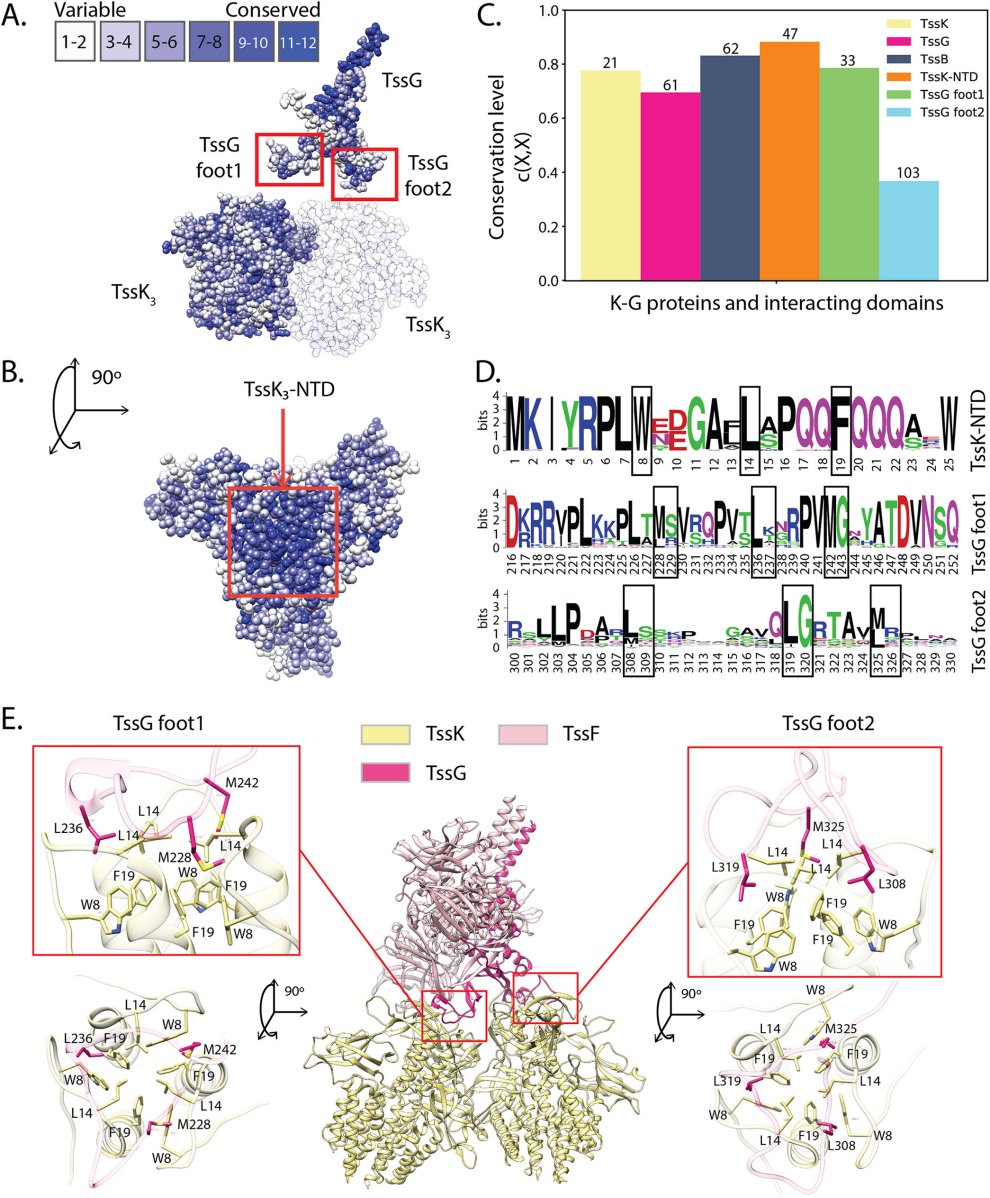
EAEC TssK-TssG interface conservation. (A and B) TssK and TssG residue conservation obtained with aligned EAEC variants mapped on the EAEC wedge complex structure (PDB 6N38). A dense group of highly conserved residues can be observed on the top of TssK trimers. This is particularly visible in panel B, in the top view of the trimer. TssG exhibits another group of conserved residues on its antenna (A), which is the domain involved in the interaction with the two TssF copies of the wedge complex. (C) Conservation level for TssK, TssK NTD, TssG, TssG foot 1, TssG foot 2, and TssB on EAEC. The numbers at the top of the bars indicate the amount of aligned EAEC homolog sequences used to compute the conservation level. (D) Sequence logos illustrating the residue conservation of the TssK-TssG interacting regions for EAEC close homologs. The height of each letter represents the information content of the corresponding amino acid at that position in bits. Black boxes highlight the three hydrophobic residues forming the TssK NTD hydrophobic cavity on top of TssK trimers and the TssG foot LG repeats. (E) TssK-TssG binding sites. The triangular TssG loops, foot 1 and foot 2, bind TssK trimers on its N-terminal region. These contacts are mediated by hydrophobic interactions between the TssK NTD hydrophobic cavity and the conserved LG repeats of both feet.

Based on the role of TssG feet in T6SS biogenesis, we hypothesized that targeting the interaction between TssK and TssG through a suitably designed molecule would essentially reproduce the results of TssG manipulations and hence would be detrimental to T6SS function. For this purpose, we decided to perform an in-depth analysis of the TssK-TssG interface and identify critical motifs that can guide toward the rational design of a biomimetic peptide (52). To better decipher the EAEC TssK-TssG binding region and to highlight molecular properties required for its interference, we conducted a conservation study using MSAs of EAEC close homologs. TssK is a trimeric protein composed of three domains: an N-terminal β sandwich shoulder domain (NTD), a 4-helix bundle middle domain, and a C-terminal α/β head domain (CTD) (46). Mapping the amino acid conservation onto the TssK and TssG structures revealed a clustering of highly conserved motifs located on the TssK NTD (Fig. 2A and B). Remarkably, the TssK NTD has a conservation level that is higher than that of the full-length TssK and TssG proteins, as well as TssG foot 1 and TssG foot 2, and even exceeds the conservation level of TssB protein, which is one of the most conserved proteins in T6SS (13) (Fig. 2C; also, see Materials and Methods). The high conservation level of the TssK NTD, which directly interacts with the TssG feet, strengthens the hypothesis that this region might serve as a target for therapeutical purposes.

**FIG 3:**
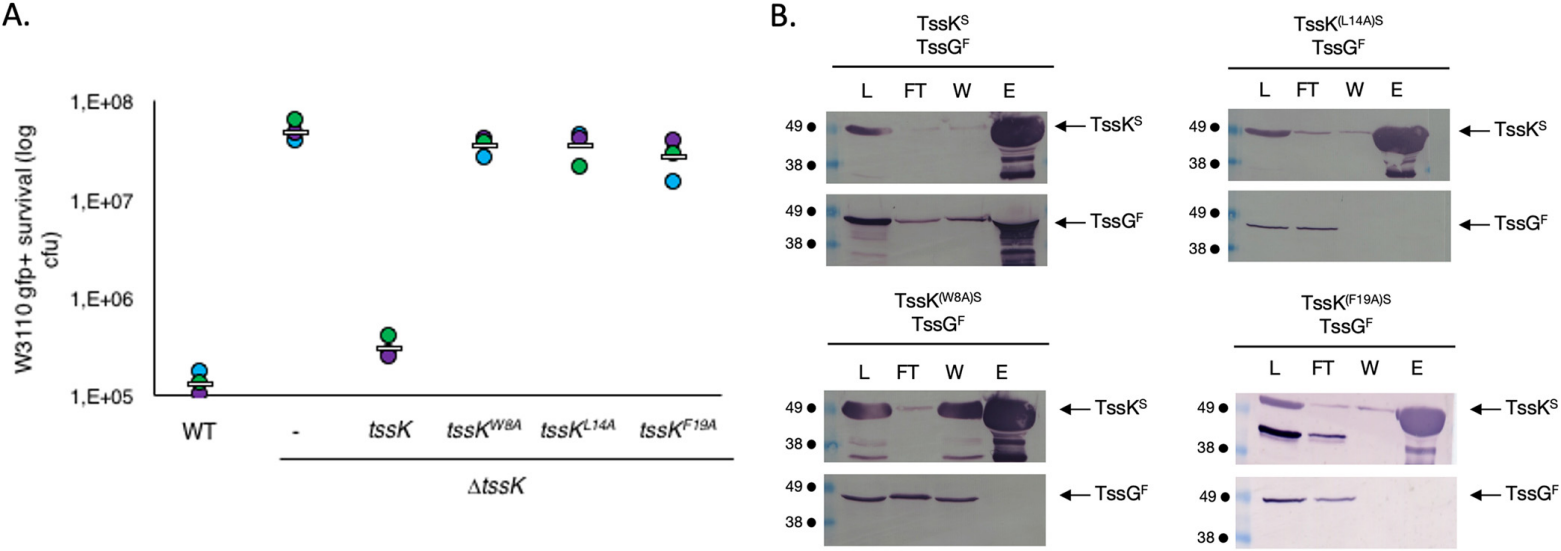
TssG foot domains are essential for T6SS functioning. (A) Antibacterial assay. The relative fluorescent level (in arbitrary units) and the number of recovered Escherichia coli recipient cells (in log_10_ CFU) are indicated. The assays were performed at least three independent times, with technical triplicates, and the measurements of a representative technical triplicate (circles) are shown, with the corresponding average value calculated for all assays (white bar). (B) Interaction between Strep-tagged TssK (TssK^S^) and Flag-tagged TssG (TssG^F^) studied by affinity copurification. Copurification was carried between TssG^F^ and variants of TssK^S^. TssK harboring the mutations W8A, L14A, and F19A are indicated by TssK^(W8A)S^, TssK^(L14A)S^, and TssK^(F19A)S^, respectively. Soluble extracts of E. coli BL21(DE3) cells producing the proteins indicated on top of the gels were submitted to an affinity purification step on a StrepTrap column, pulling down Strep-tagged TssK. The lysate (total soluble material [L]), flowthrough (FT), wash (W), and eluate (E) were subjected to denaturation by 12.5% acrylamide PAGE and subsequently immunodetected with the appropriate antibody. Immunodetected proteins are indicated on the right, while molecular weight markers (in kDa) are indicated on the left. As observed for wild-type TssK^S^, the simultaneous presence of a band in the eluate of TssK and a band in the eluate of TssG indicates the presence of interaction between the two proteins. As observed for all three TssK^s^ mutants, the presence of a band in the eluate of TssK and the absence of a band in the eluate of TssG indicate the lack of interaction.

TssG foot 1 has a conservation level lower than that obtained for the TssK NTD, although similar to that of the full-length TssK, while foot 2 is very variable. Nevertheless, both feet possess a conserved repeated pattern consisting of a hydrophobic amino acid (e.g., leucine or methionine) followed by a small or a basic residue (e.g., glycine, serine, arginine, or lysine) alternating with a variable region, i.e., LGXXXX^1^LGXXXX^2^LG (referred to here as the LG repeat). The first variable region (XXXX^1^) is heterogeneous in length and composition, while the second (XXXX^2^) has a conserved length of about four residues, and it is less variable (Fig. 2D). Substitution of the three hydrophobic residues in the TssG LG repeat motif by an arginine in EAEC has been shown to impair TssK binding *in vitro* (51). This observation indicates that the interaction between TssK and TssG is stabilized by hydrophobic interplays between the conserved LG repeats of both TssG feet and the TssK NTD. In agreement with these observations, the TssK NTD harbors three highly conserved amino acids (W8, L14, and F19) forming hydrophobic cavities hosting the three LG repeat motifs in each TssG loop (Fig. 2D and E). To analyze the role of these three interfacial motifs, we performed alanine-scanning mutagenesis and created TssK variants (i.e., W8A, L14A, and F19A). In EAEC, a TssK transcomplementation experiment with a plasmid expressing each TssK mutant did not restore T6SS-dependent killing of the prey (Fig. 3A). Biochemically, these three mutations had no impact on the stability or the oligomeric state of TssK (Fig. S1E) but abolished the direct interaction with TssG, as monitored by affinity copurification experiments where streptavidin (Strep)-tagged TssK variants were used to pull down TssG (Fig. 3B). We thus concluded that the TssG-TssK hydrophobic interaction is an absolute determinant for the entire wedge complex stability and is also highly specific, since a mutation of each of the three side chains belonging to the hydrophobic pocket of TssK leads to the complete disruption of the assembly.

### Design of a biomimetic cyclic peptide.

**FIG 4:**
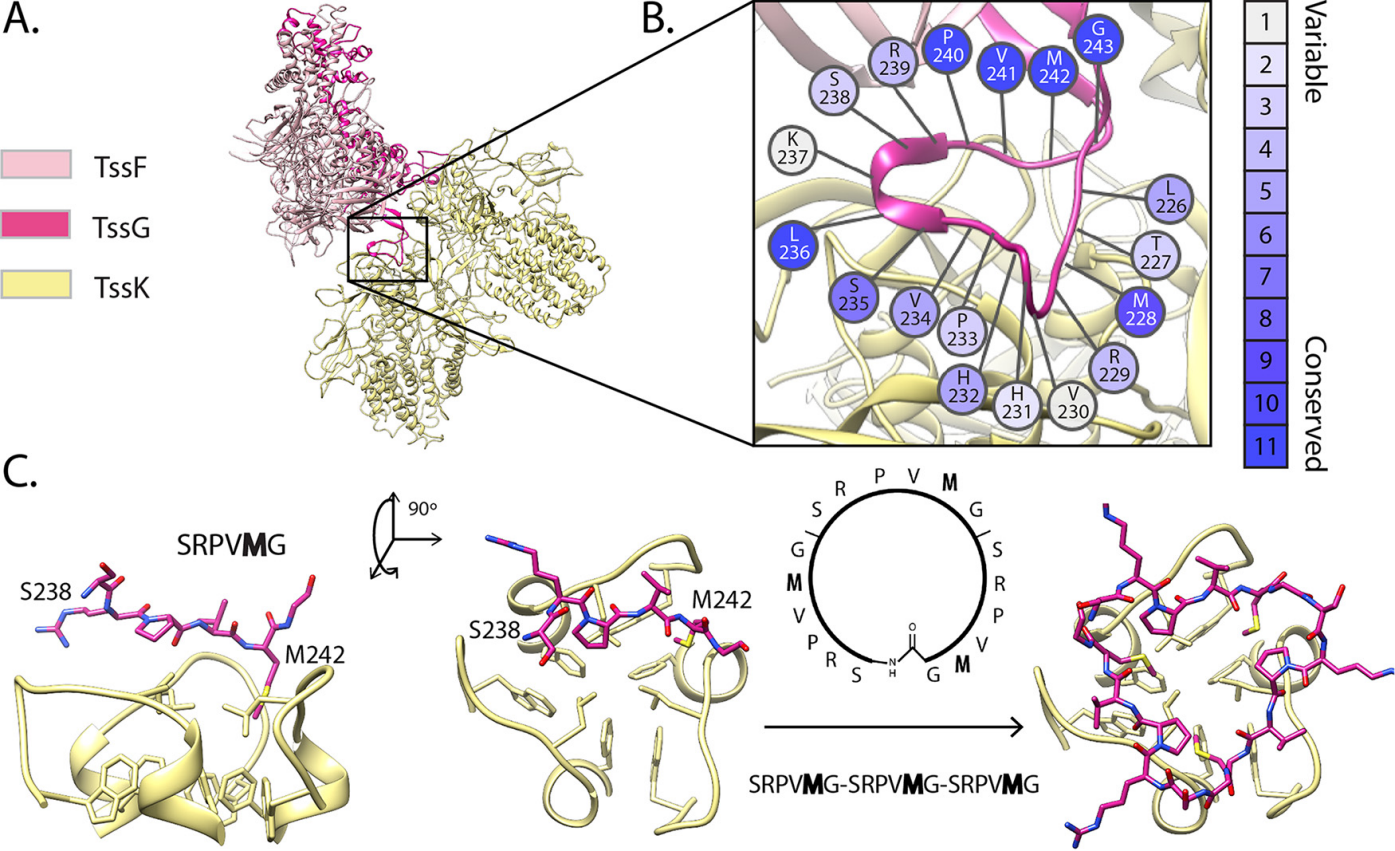
Biomimetic inhibitor *in silico* design. (A) EAEC wedge complex (PDB ID 6GIY). The box indicates the position of TssG foot 1, the template used to design the inhibitor. (B) The residue conservation of TssG foot 1 calculated from EAEC variants was used together with other indicators, such as coevolution, to select the biomimetic candidate segment (see Materials and Methods). (C) A 6-residue peptide of foot 1 (from S238 to G243) was used to generate a cyclic peptide that mimics foot 1 in its interaction with TssK trimer.

Inspired by the specific TssK-TssG hydrophobic interaction deciphered above and its influence on the T6SS function, we aimed to design a peptide inhibitor mimicking the arrangement of the TssG foot domains bound to TssK. Structural analysis of the TssKFGE unit revealed that the TssG feet region harbors an intrinsic triangular fold that fits the C3-symmetric shape of the TssK NTD (Fig. 4). Following this observation, we hypothesized that a peptide with a cyclic structure and LG repeats organized as in TssG feet might be sufficient to bind to the TssK NTD and hence compete with TssG association (Fig. 4). We decided to design such a peptide using the TssG foot 1 as a template for two main reasons: (i) foot 1 has a higher conservation level than foot 2 (Fig. 2C) and (ii) foot 1 is less structurally complex than foot 2. In fact, foot 2 has a partially globular fold and more intramolecular contacts than foot 1. Based on the residue conservation analysis (Fig. 4B), we selected the sequence between the second and the third LG repeats of TssG foot 1, SRPVMG (positions 238 to 243), as a repeat unit. The SRPVMG unit was repeated twice to fill the three hydrophobic cavities located within the TssK NTD (Fig. 4C), resulting in the sequence SRPVMG-SRPVMG-SRPVMG. We applied the head-to-tail cyclization to create a triangular shape that could best fit the TssK trimeric fold (Fig. 4C). In parallel and to further substantiate the inhibitory potential of the biomimetic cyclic peptide (BCP), we rationally designed two additional cyclic peptides for control experiments (Fig. S8A): (i) a randomized version (SVRMPG-SPMRVG-SMVRPG) (see Materials and Methods) aiming to evaluate the importance of the amino acid order in the binding to TssK NTD and (ii) a mutated version generated by mutating each methionine to alanine (SRPVAG-SRPVAG-SRPVAG) to explore the importance of the conserved methionine residue in binding to the TssK hydrophobic pocket.

10.1128/mBio.01348-21.8 FIG S8 (A) Design of the randomized BCP. The 13,824 cyclic peptide candidates for the negative-control experiments are ranked according to their median total energy (dark blue line) of 100 minimized snapshots obtained from MD simulations. For each candidate, the light blue vertical line represents the first and third quartile of the 100 total energies. The yellow, black, and red stars show the ranking of the BCP (6th), the mutated BCP (161st), and the randomized BCP (12,203rd), respectively. (B) Isothermal calorimetry was used to demonstrate the specific interaction between TssK and BCP. The figure shows the interaction between 25 μM TssK-WT and 1 mM WT (green) or mutant BCP (red). (Top) Heat exchange upon TssK ligand titration with BCP. (Bottom) Integrated data with binding isotherms. Raw data are shown in closed circles, and a 1:1 fit is shown in a solid line. Download FIG S8, TIF file, 1.7 MB. Copyright © 2021 Cherrak et al. 2021 Cherrak et al. https://creativecommons.org/licenses/by/4.0/ This content is distributed under the terms of the Creative Commons Attribution 4.0 International license.

### Molecular properties of the BCP and specificity of its binding to TssK.

In order to characterize the biophysical and binding properties of the peptide, a series of nuclear magnetic resonance (NMR) experiments were performed. These experiments included one-dimensional (1D) ^1^H-^1^H total correlation spectroscopy (^1^H-^1^H-TOCSY), ^1^H nuclear Overhauser effect spectroscopy (^1^H-NOESY), and ^1^H-^15^N heteronuclear single quantum coherence (HSQC) spectroscopy using its ^15^N natural abundance. ^1^H-^15^N HSQC spectroscopy is of particular interest in characterizing the peptide, as each H/N correlation peak is associated with the NH group of an amino acid in a particular chemical and magnetic environment. Here, we observed only 5 correlations for the 5 observable amino acids out of 6 (Fig. S7A), the last being a proline. This confirmed that the amino acids of the three repeated (circular) sequences are equivalent (i.e., 3-fold axis symmetry). In addition to HSQC spectroscopy, the ^1^H-NOESY experiment did not exhibit any indications of a particular 3D folding (Fig. S7B). After addition of a 1:10 final concentration of TssK (1 part TssK to 10 parts BCP), the ^1^H-^15^N HSQC correlations (same conditions as the free-peptide experiment) totally disappear, while 90% of the peptide is supposed to be in the unbound form, in solution. The ^1^H-^15^N HSQC spectrum could not be recovered by a longer data accumulation. This deleterious effect was also confirmed on the 1D ^1^H proton NMR spectrum (Fig. S7C), exhibiting a strong broadening of the peptides peaks that clearly indicates information transfer between the peptide and the protein either by chemical exchange relaxation (millisecond range for *k*_on_/*k*_off_) (*k*_on_ [association rate constant] is the rate at which an interaction happens per second in a unimolar mixture; *k*_off_ [dissociation rate constant] is the fraction of complex which dissociates per second) or a peptide/protein size cross-relaxation effect. Overall, these experiments confirmed the absence of an internal structure of the free peptide and confirmed our *in silico* prediction.

10.1128/mBio.01348-21.7 FIG S7 Cyclic peptide NMR characterization. (A) Natural ^15^N abundance ^1^H-^15^N HSQC spectrum of the cyclic peptide. (B) ^1^H-NOESY spectrum of the cyclic peptide. (C) Superimposition of the 1D ^1^H cyclic peptide (blue) to the peptide-TssK complex (10:1 concentration ratio) spectrum (red). Download FIG S7, TIF file, 2.2 MB. Copyright © 2021 Cherrak et al. 2021 Cherrak et al. https://creativecommons.org/licenses/by/4.0/ This content is distributed under the terms of the Creative Commons Attribution 4.0 International license.

**FIG 5:**
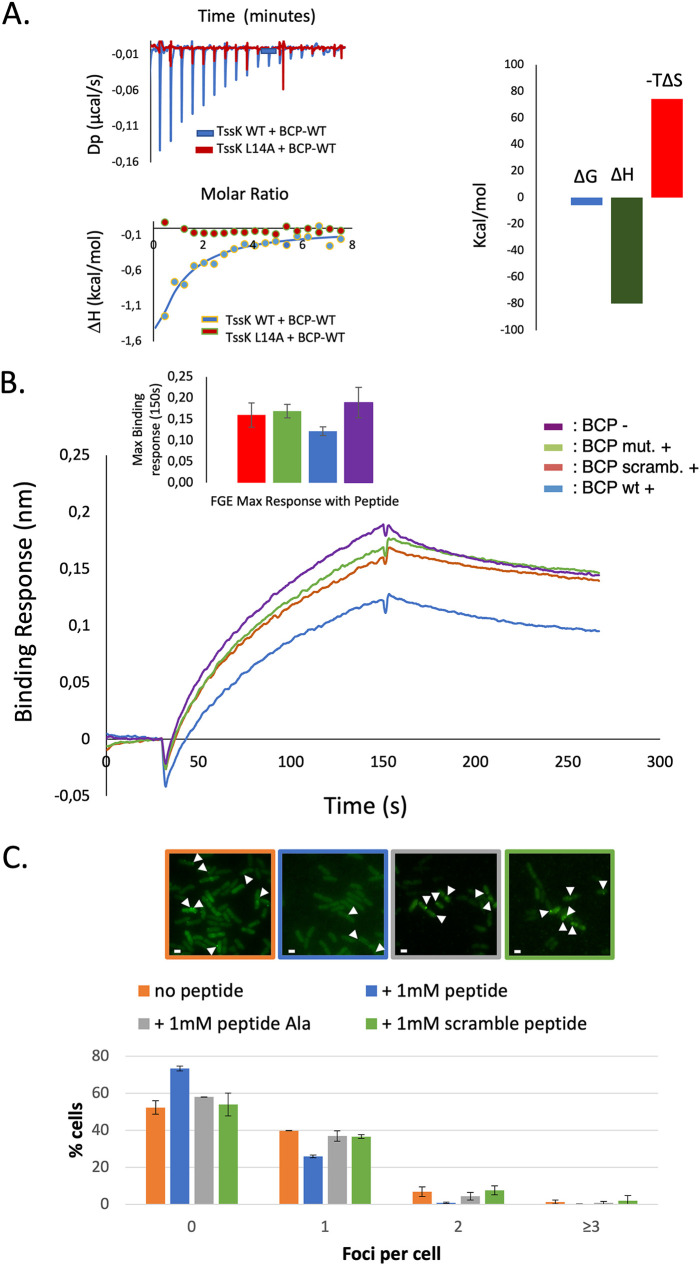
Biomimetic peptide interfere with TssK-TssG interface. (A) Isothermal calorimetry was used to demonstrate the specific interaction between TssK and wild-type BCP. (Left) Interaction between 25 μM TssK-WT, TssK-L14A and 1 mM wild type BCP. (Top) Heat exchange upon TssK ligand titration with BCP for TssK WT as ligand and BCP as analyte (blue curve), and for TssK-L14A as ligand and BCP as analyte (red curve). (Bottom) Integrated data with binding isotherms (solid line) fitted to a single-site binding model. The constant heat dilution was removed before the integrated binding isotherms. The red squares indicate the integrated data of TssK-WT and BCP, and the green triangles show the lack of binding for TssK L14A and BCP. (Right) Signature plot of TssK-WT with BCP, suggesting an enthalpy-driven interaction with a favorable Δ*G*. (B) TssF-TssG-TssE binding to biotinylated TssK. Binding sensograms display responses from three independent assays of 47 μM TssFGE binding to TssK in the presence or absence of different 1 mM BCP constructs in kinetic buffer. Each sensogram represents three steps: initial baseline, association, and dissociation. Top to bottom: delta BCP (purple), mutated BCP (green), randomized BCP (red), and wild-type BCP (blue). (Inset) Maximum association response at 150 s of TssFGE binding to TssK in the presence or absence of different BCP constructs. The standard deviation is computed over three independent assays. Left to right: randomized BCP (red) (0.160 ± 0.028 nm), mutated BCP (green) (0.169 ± 0.016 nm), wild-type BCP (blue) (0.121 ± 0.010 nm), and delta BCP (purple) (0.189 ± 0.036 nm). (C) Biomimetic peptide diminishes TssK-sfGFP foci *in vivo*. EAEC with a TssK-sfGFP chromosomic fusion was subjected to a hypo-osmotic shock in the presence of 1 mM peptide or in the presence of buffer for the control. After regrowing, cells were observed by microscopy. (Top) Fluorescence microscopy illustrations of TssK-sfGFP foci with or without the peptide and control peptides (mutated and randomized). Examples of foci corresponding are indicated by arrowheads. Bar, 1 μM. (Bottom) Focus quantitation from three independent experiments. The BCP decreased TssK-sfGFP foci compared to both control peptides. The experiment was done in triplicate, and ∼500 cells were analyzed for each condition.

To evaluate the direct interaction between the BCP and its target TssK, we performed isothermal titration calorimetry (ITC) experiments. The wild-type BCP and TssK interact with an observable affinity (*K_D_*) of 60 μM (Fig. 5A). The ITC binding signature of TssK-BCP suggests that it is driven by a favorable enthalpy, presumably based on hydrogen bonds and van der Waals interactions. The positive entropy suggests conformational changes which could be responsible for the low affinity observed. To confirm the specificity of this interaction, we performed a series of controls consisting of (i) mixing the BCP with the TssK-L14A variant, which we previously demonstrated has lost its capacity to interact with TssG, and (ii) incubating TssK with each of the control peptides. None of these conditions led to a detectable ITC binding signal (Fig. 5A and Fig. S8B), demonstrating the direct liaison of BCP to the TssK target, precisely at the TssG foot binding site.

### Inhibition of the EAEC TssK-TssG interface by the BCP.

We conducted *in vitro* and *in vivo* experiments to assess the potential T6SS-blocking activity of our BCP. First, the assembly of the TssKFGE wedge complex was reconstituted *in vitro* on the surface of a biolayer interferometry biosensor (Fig. 5). Briefly, biotinylated TssK trimers were immobilized onto a biosensor. Normalized bacterial cell lysate without TssFGE plasmid was added to the TssK-bound biosensor to block free Strep sites. To demonstrate the specific binding of TssFGE with TssK, normalized bacterial cell lysate expressing the TssFGE protein partners was added to the bound TssK to form the TssKFGE wedge complex (Fig. 5B and Fig. S2). TssK was shown to interact specifically with its natural partners, with a maximum binding average response from three independent assays of 0.189 ± 0.036 nm at 150 s association. When TssK was preincubated with the different BCP constructs, i.e., the mutated BCP, the randomized BCP, and the wild-type BCP, the maximum binding average responses of three independent assays were 0.169 ± 0.016 nm, 0.160 ± 0.028 nm, and 0.121 ± 0.0102 nm, respectively (Fig. 5B, inset), demonstrating that the wild-type BCP significatively interferes with the TssKFGE formation and the control peptides have a negligible activity.

10.1128/mBio.01348-21.2 FIG S2 (A) Biomimetic peptide diminishes TssFGE binding. The 50 μM TssFGE lysate control signal without bound TssK was subtracted from that for both TssK-TssFGE or TssK-BCP-TssFGE binding signal (Fig. 5). the sensogram displays a decreasing binding response over time in the presence of the BCP. The gray shade represents the standard deviation computed over three independent experiments. (B) BLI TssK mean loading curve. Streptavidin biosensors were loaded with biotinylated TssK for 120 s, with an average loading response of 1 nm. (C) TssF, TssG, and TssE were present in the TssFGE lysate but not in the control lysate. TssFGE and control lysate protein expression was verified by 12.5% SDS-PAGE and immunoblotting. TssF was detected with anti-Strep, TssG with anti-Flag, and TssE with anti-HA primary antibodies and revealed by anti-mouse antibody coupled to alkaline phosphatase. Download FIG S2, TIF file, 2.2 MB. Copyright © 2021 Cherrak et al. 2021 Cherrak et al. https://creativecommons.org/licenses/by/4.0/ This content is distributed under the terms of the Creative Commons Attribution 4.0 International license.

### Modulation of bacterial competition by the BCP.

Based on the BCP ability to bind to TssK and to limit the *in vitro* binding of the TssFGE wedge components onto TssK, we evaluated its T6SS interference potential on bacteria. We set up a competition experiment between EAEC (predator, T6SS^+^) and E. coli (prey, T6SS^−^) strains (see Materials and Methods) in the presence or absence of the BCP. Incubation of cells with 1 mM BCP is accompanied by a drastic decrease of prey cells number, similar to what is observed in the absence of BCP. We concluded that the BCP does not affect the killing ability of EAEC when added directly in the competition mixture (Fig. S3C), which can be explained by its physicochemical properties, which prevent its diffusion across biological membranes. To test this hypothesis, we developed a protocol favoring BCP entry into bacterial cells without any electroporation or chemical steps likely to impair T6SS activity (see Materials and Methods). This procedure, relying on an osmotic change, has been applied to the TssK-sfGFP strain, which allowed us to enumerate the number of fluorescent foci and hence assembled baseplates (45, 50) in presence or absence of the BCP (Fig. 5C). Osmoporation of the TssK-sfGFP strain with 1 mM BCP increased significantly (∼30%) the proportion of bacteria with no observable foci compared to peptide-free treated cells (Fig. 5C). In contrast, incubation with a 1 mM concentration of the control peptides did not impact the number of fluorescent foci, which was comparable to that in the free-peptide condition. Importantly, the peptide was not toxic for bacteria up to 1 mM (Fig. S3A), and the osmotic stress did not affect fluorescent-focus assembly (Fig. S3B).

10.1128/mBio.01348-21.3 FIG S3 (A) The peptide exhibits no cell toxicity EAEC with TssK-GFP fusion was grown in SIM medium with or without 1 mM BCP, and optical density at 600 nm was monitored in a spectrofluorimeter. The BCP had no impact on the growth of EAEC. Results are the means for biological triplicates. (B) The hypo-osmotic shock is mandatory for BCP entry into cells. EAEC with the TssK-GFP fusion was grown in SIM medium to favor T6SS expression. Cells were then subjected to hypo-osmotic shock induced by NaCl, diluted, and recultured to induced T6SS re-expression. At the appropriate OD at 600 nm, cells were observed under the microscope, and foci were counted. The hypo-osmotic shock does not drastically impact the ability of TssK-GFP to form foci. TssK-GFP foci in cells without osmotic shock are comparable to those without peptide. (C) Antibacterial growth competition assay. Prey bacteria (E. coli K-12 strain W3110 bearing the pUA66-rrnB plasmid) and attacker bacteria (EAEC T6SS^+^ and EAEC T6SS^−^) were mixed in the presence or absence of the BCP. The survival of the prey bacteria was monitored by CFU enumeration. Download FIG S3, TIF file, 2.2 MB. Copyright © 2021 Cherrak et al. 2021 Cherrak et al. https://creativecommons.org/licenses/by/4.0/ This content is distributed under the terms of the Creative Commons Attribution 4.0 International license.

### Evolution of the TssK-TssG interaction interface.

Our functional analysis and peptide design revealed the determinants of the TssK-TssG interaction, which relies on hydrophobic interplays conserved within EAEC. To assess whether BCP can target other T6SS-harboring bacteria, we systematically compared the sequences of full-length TssK, TssG, TssK-TssG interacting domains (i.e., the TssK NTD and TssG foot 1 and foot 2), and TssB, which was used as a reference. For this purpose, we (i) collected the sequences from bacteria encoding a T6SS directly linked to pathogenicity or with host-associated activity (Table S1B and C), (ii) clustered the sequences based on the sequence similarity and also their variants, and (iii) computed their conservation levels.

10.1128/mBio.01348-21.10 TABLE S1 (A) Strains, plasmids and oligonucleotides used in this study. (B) Bacterial strains naming for the *in silico* approach. (C) Protein sequences for the *in silico* approach. Download Table S1, DOCX file, 0.04 MB. Copyright © 2021 Cherrak et al. 2021 Cherrak et al. https://creativecommons.org/licenses/by/4.0/ This content is distributed under the terms of the Creative Commons Attribution 4.0 International license.

**FIG 6:**
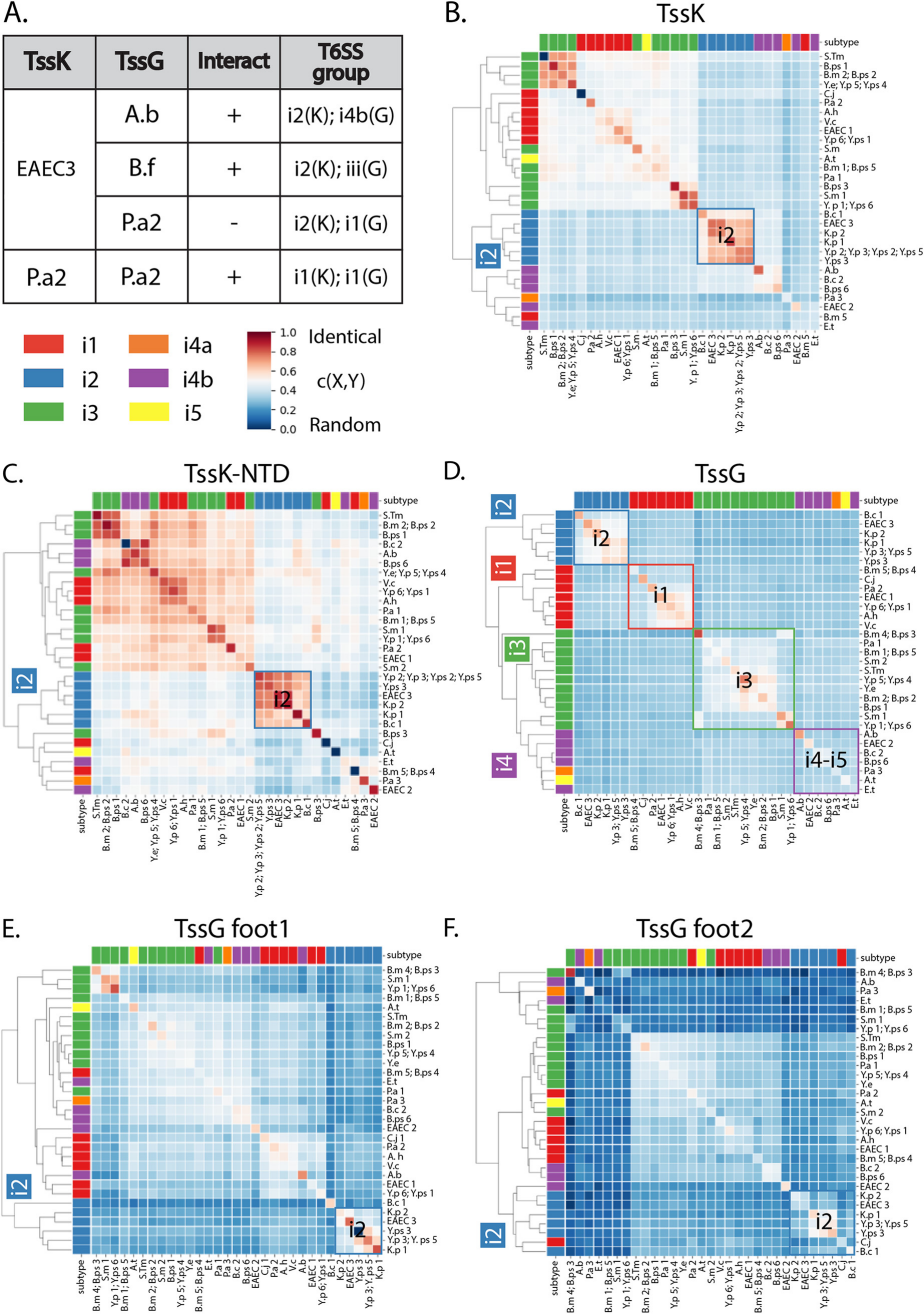
Phylogeny and conservation of the TssK-TssG interface among pathogenic bacteria. (A) The TssK-TssG interface shares common features among pathogenic bacteria. Summary of the results of copurification experiments presented in detail in Fig. S5. EAEC3 or P.a2 TssK was tested for the interaction with A.b, B.f, or P.a2 TssG. A positive interaction is reported with a plus sign, whereas a negative interaction is reported with a minus sign. T6SS subtypes are indicated in the right column for each pair. (C to F) Hierarchical clustering of TssK, TssG, and corresponding interacting domains using the closeness metric (see Materials and Methods). Diagonal values represent the conservation level of a protein or a domain for a given pathogen, while out-of-diagonal values represent the similarity of a protein or a domain calculated between two different pathogens. Gene cluster nomenclature is fully reported in Table S1A. For example, EAEC1 represents the first T6SS gene cluster found in the EAEC genome, while EAEC3 is the third. Clusters of pathogens that follow the standard subtype classification for T6SS are indicated by the color of the matching square.

Unexpectedly, TssK and TssG have divergent sequence clustering patterns, while being structural neighbors (Fig. 6). Indeed, clustering of TssB and TssG sequences (Fig. 6D) followed the standard T6SS subtype classification (53) (Fig. S6B), whereas TssK (Fig. 6B) did not. i2 is the only T6SS subtype that is consistently clustered in both TssK and TssG as well as their interacting domains. Importantly, our EAEC T6SS model (referred to here as EAEC3 to differentiate the three EAEC T6SS gene clusters under study) belongs to this specific T6SS subtype. This pattern indicates the existence of a conserved and specific TssK-TssG interface signature and suggests that the interfering activity spectrum of the BCP applies not only to EAEC3 but also to other subtype i2 T6SSs, such as those of Klebsiella pneumoniae (K.p 1 and K.p 2), Yersinia pseudotuberculosis (Y.ps 3 and Y.ps 5), Yersinia pestis (Y.p 3), and Burkholderia cenocepacia (B.c 1) (Table S1A; Fig. S6C).

10.1128/mBio.01348-21.6 FIG S6 (A) Sequence logos representing the residue conservation of TssK-TssG interacting domains for all pathogens. These sequence logos were computed using not only EAEC variants but also TssK and TssG sequences from EAEC distant T6SS. Black boxes highlight the residues forming the hydrophobic cavity on the top of TssK trimers and the LG repeats on TssG feet. (B) Hierarchical clustering of TssB using the closeness metric. Diagonal values represent the TssB conservation level for each pathogen. Out-of-diagonal values represent the TssB similarity level between different pathogens. The color code on the left and top of the heat map indicates the T6SS subtype classification, which matches the standard one (53). (C) TssK NTD, TssG foot 1, and TssG foot 2 multiple-sequence alignment for subtype i2 pathogens. Download FIG S6, TIF file, 1.7 MB. Copyright © 2021 Cherrak et al. 2021 Cherrak et al. https://creativecommons.org/licenses/by/4.0/ This content is distributed under the terms of the Creative Commons Attribution 4.0 International license.

10.1128/mBio.01348-21.5 FIG S5 (A) Purification experiment controls. Soluble extracts of E. coli BL21(DE3) cells producing the TssG-Flag-tagged protein from the indicated strain were subjected to an affinity purification step on a StrepTrap to assess nonspecific protein binding. (B) The TssK-TssG interface shares common features among pathogenic bacteria. Soluble extracts of E. coli BL21(DE3) cells producing the indicated proteins from the indicated strain were subjected to an affinity purification step on a StrepTrap. (C) Soluble extracts of E. coli BL21(DE3) cells producing the TssK variant mutated at position 8 (W8A) with the indicated TssG protein were subjected to an affinity purification step on a StrepTrap. (D) Reciprocal copurification experiment. TssK-Strep from B. fragilis and A. baumannii were used to copurify the EAEC TssG. The lysate (total soluble material [L]), flowthrough (FT), wash (W), and eluate (E) were subjected to denaturing 12.5% acrylamide PAGE and immunodetected with the appropriate antibody. Immunodetected proteins are indicated on the right. Molecular weight markers (in kDa) are indicated on the left. S, Strep; F, Flag. Download FIG S5, TIF file, 1.7 MB. Copyright © 2021 Cherrak et al. 2021 Cherrak et al. https://creativecommons.org/licenses/by/4.0/ This content is distributed under the terms of the Creative Commons Attribution 4.0 International license.

The overall sequence conservation level was computed for the full-length TssK, TssG, and TssB proteins as well as the interacting domains TssK NTD, TssG foot 1, and TssG foot 2 of each pathogen (Fig. S4 and Fig. 2C). In general terms, the TssK NTD has a higher conservation level than full-length proteins and TssG feet. TssG foot 2 is less conserved than foot 1. Multiple-sequence alignments of TssK NTD for all pathogens and their variants (2,482 sequences) show that the TssK NTD hydrophobic amino acids W8, L14, and F19 are highly conserved, as they are for EAEC3 (Fig. S6A). Likewise, considering the MSA for all pathogens (3,712 sequences), the LG repeat motif is consistently conserved in both feet (Fig. S6A). All these observations indicate that all the TssK-TssG interfaces of pathogenic T6SS follow the same physicochemical rules as in EAEC3, which consists of TssK hydrophobic cavities accommodating the TssG hydrophobic residues. Based on this analysis, we therefore expect that the BCP can bind to a larger repertoire of TssK targets and hence have a greater spectrum of activity.

10.1128/mBio.01348-21.4 FIG S4 For all gene clusters isolated from all considered pathogens, the conservation level of TssK, TssK NTD, TssG, TssG foot 1, TssG foot 2, and TssB was computed. Each plot has a label that indicates the name of the pathogen, the number of the T6SS gene cluster, and the T6SS subtype. For example, “Y.ps 2 – i2” stands for Yersinia pestis cluster 2, subtype i2. Download FIG S4, TIF file, 1.7 MB. Copyright © 2021 Cherrak et al. 2021 Cherrak et al. https://creativecommons.org/licenses/by/4.0/ This content is distributed under the terms of the Creative Commons Attribution 4.0 International license.

### Proof-of-concept evaluation of the broad-spectrum activity of the anti-T6SS BCP.

Based on the high and low conservation levels, respectively, of the TssK NTD and TssG feet, we hypothesized that the TssK-TssG hydrophobic interplay in EAEC3 is a general binding mechanism. To challenge this hypothesis, we investigated the ability of the EAEC TssK target to accommodate several TssG protein partners encoded in strains clustered outside the T6SS i2 subtype. We selected three TssG homologs belonging to different subgroups (Fig. 6A), including the pathogenic species Acinetobacter baumannii (A.b subgroup i4b) and Pseudomonas aeruginosa H2 (P.a2 subgroup i1) as well as a member of the human microbiota, Bacteroides fragilis (B.f subgroup iii). We performed protein copurification experiments with TssK-TssG pairs and evaluated cross-species interactions. No interaction was observed between TssK_EAEC_ and TssG*_Pa_*, while TssK_EAEC_ interacts with both TssG*_Bf_* and TssG*_Ab_* (Fig. 6A and Fig. S5B), and reciprocally, TssG_EAEC_ interacts with both TssK*_Bf_* and TssK*_Ab_* (Fig. S5D). To validate these interactions, we performed the same experiments using the EAEC TssK variant mutated at the W8 residue. Substitution of the TssK hydrophobic residue tryptophan by an alanine induces the loss of interaction with TssG*_Bf_* and TssG*_Ab_*, suggesting that these proteins interact through a mechanism similar to that in EAEC (Fig. S5C). Based on the interaction between TssK_EAEC_ and TssG homologs from non-co-occurring species, we proposed that the TssK-TssG binding properties expands beyond the i2 subtype. Overall, the high conservation of hydrophobic characteristics found in both protein sides combined with biochemical experiments revealed that few determinants are required to drive the interaction specificity between TssK and TssG. We thus propose that BCP provides a framework for the design of broad-spectrum T6SS inhibitors targeting multiple bacterial pathogens.

## DISCUSSION

The T6SS is a phage-related contractile machinery responsible for toxin secretion into both eukaryotic and prokaryotic cells. This secretion apparatus is found in more than 25% of Gram-negative bacteria, including the ESKAPE pathogens K. pneumoniae, A. baumannii, P. aeruginosa, and Enterobacter species, where it plays a significant role in either colonizing and damaging the host or manipulating and evading the immune system (12, 23–27, 54–57). Although the T6SS is structurally well characterized and meets all the criteria as a promising virulence factor candidate, a very limited number of studies have attempted to develop anti-T6SS molecules or peptides. Certain studies have used phenotypic screening (58), and others have employed a targeted approach, such as blocking the interaction between the two sheath proteins (TssB and TssC) or the dissociation of a toxin immunity complex (28, 59). Another study employed a nanobody intracellularly expressed to block the assembly of the membrane complex (MC) (60), which is limited in terms of a possible therapeutic application, since nanobodies cannot cross the bacterial outer membrane. All these studies lack a molecular and structural description of the hit binding site, which precluded hits-to-lead optimization and a broad applicability to a large number of human pathogens, including the ones from the ESKAPE list. In our study, we decided to combine structural, molecular, and bioinformatics analysis with the aim of targeting a conserved molecular interface in the T6SS baseplate that is crucial for the functioning of the whole nanomachine.

The TssK-TssG interface plays a pivotal and primordial role in the early stages of the T6SS baseplate assembly, specifically, the two small unfolded TssG domains interacting with the N-terminal region of TssK (50, 51). Using BACTH experiments, previous studies demonstrated the total loss of interaction between TssK and TssG variants mutated in either the foot 1 or foot 2 domain, suggesting that TssG requires two functional feet to bind to TssK (51). In our study, we demonstrated that deletion of either TssG foot 1 or foot 2 abrogates the formation of the TssKFGE wedge complex and is associated with a loss of T6SS activity (Fig. 1), which revealed early-stage T6SS baseplate assembly as a promising target. Overall, we provide strong evidence that TssG foot domains are essential for T6SS activity and bind to key hydrophobic residues representing a docking surface on the TssK NTD. Interfering with this recognition step can be envisaged to inhibit T6SS functioning through a peptide mimicking one of the two TssG feet with hydrophobic characteristics required for TssK cavity binding.

The role of unstructured loops in macromolecular complex stability was reported previously. For instance, the T4 phage TssG homolog gp7 protein connects the tail fibers (gp9, gp10, and gp11) to the baseplate, allowing the communication of important structural transitions. During contraction, a loop formed by residues 841 to 862 of gp7 performs a “jump-rope”-type motion transferring the signal received from the fibers to the gp25-(gp6)_2_-gp7-like wedge module responsible for the conformational switch (49). In our T6SS model, TssG harbors two unfolded domains. The TssG foot 1 domain is a loop that is structurally independent from the rest of the protein. In contrast, TssG foot 2 is a partially folded region characterized by less structural autonomy. Multiple-sequence alignments of TssG in EAEC and other T6SS-harboring pathogens indicated a higher conservation level of the TssG foot 1 than TssG foot 2. Besides these differences, both TssG feet impact T6SS activity to the same extent, which suggests that they are good candidates for inhibition strategies.

While a number of studies have targeted α-helix and β-strand epitopes to block specific protein-protein interaction interfaces (PP2I) (52, 61), loop-mimicking PP2I inhibitors represent an enormous challenge, and very few bioinformatics tools have been designed to help in this quest (62). The inhibition of a protein-protein interaction is challenging. It requires either a robust and costly methodology to screen thousands of compounds from a library or the rational design of interfering molecules guided by the molecular description of the target (63). Usually, targeting the interaction between TssK and TssG through a suitably designed molecule would have required the systematic screening of ligands able to bind to the target TssK, hence competing with TssG foot docking (64–66). Instead, we decided to base our rational design on the structure of the TssKFG wedge complex that we have obtained recently (50). We decided to design a peptide interfering with the T6SS activity by targeting the crucial TssK-TssG interaction. The TssK NTD is structured, has cavities, and is well conserved, revealing a hot spot-interacting region. Remarkably, the TssK NTD can interact with the two different TssG LG repeat domains, which vary in length and composition. This observation, reflected by the lower conservation level of the TssG foot domains across EAEC variants, suggests that TssK can accommodate different partners as long as they harbor a hydrophobic motif with a triangular fold. Based on these criteria, we designed a circular peptide inhibitor inspired by the EAEC TssG foot 1 domain. The biomimetic cyclic peptide binds to TssK and interferes with wedge protein TssFGE recruitment, which consequently impairs baseplate biogenesis in permeabilized EAEC cells (Fig. 5C).

We evaluated the potential spectrum of our peptide through TssK and TssG sequence analysis. The analysis of T6SS bacterial sequences revealed that our model EAEC with other i2 subtypes encoded in Y. pestis, Y. pseudotuberculosis, K. pneumoniae and B. cenocepacia and provided a first hypothetical picture of the spectrum of BCP activity. However, LG repeats and the conserved hydrophobic cavity can be found beyond the i2 subtype, and they are a general property of the TssG-TssK interaction, including that in A. baumannii and B. fragilis, which has been confirmed by biochemical cross-species interaction experiments. The human microbiota member B. fragilis encodes T6SS clusters with a significant role in gut homeostasis and colonization resistance (67–69). The potential BCP interfering activity on B. fragilis T6SS needs to be carefully considered to limit any collateral damage ensuing from antivirulence treatment. However, enterotoxigenic B. fragilis (ETBF) is involved in colorectal cancer (CRC) development (70), and more broadly, B. fragilis is found in a number of antibiotic-resistant bacterial infections (71). Consequently, targeting the B. fragilis T6SS might represent an alternative way to fight this pathogen. The bioinformatics analysis predicting the presence of LG repeats and hydrophobic cavities in the P. aeruginosa H2 TssK-TssG interface contrasted with our biochemical experiments. This unexpected observation indicates that molecular properties different from the LG repeats could additionally drive TssK-TssG specificity and highlights the need to experimentally explore and validate interface conservation across bacteria.

Several improvements can be considered for the BCP, for instance, its limited molecular diffusion in cell membranes. The BCP has a molecular weight exceeding 600 Da, which is the porin-mediated diffusion limit (72). To overcome this obstacle, we could consider fusing our BCP with either siderophores (73) or cell-penetrating peptides (CPPs). Examples of CPP-aided delivery include an inhibitor of bacterial FtsZ altering the growth of S. aureus (74). Implementation of permeation rules increasing drug spectrum activity toward Gram-negative bacteria can be an alternative to rationally modifying BCP diffusion properties (75). In addition to its low diffusion property, the inhibitory effect of the peptide has been tested with a relatively high working concentration (1 mM). This, coupled with our observation that the BCP effect is maximum within the first hour after peptide entry, suggests that both its low affinity and stability are important to consider in the framework of future improvements. In spite of these limitations, we think that the BCP can be used as a scaffold to design a class of clinically relevant inhibitors with increased diffusion properties, better affinity for TssK, and improved activity against T6SS function.

In general, this work provides an in-depth understanding of the EAEC TssK-TssG interface, which has been proven to be a promising target for T6SS inhibition. We highlighted molecular determinants required for the TssK-TssG interference that guided the rational design of a biomimetic peptide for which the activity against EAEC validated the feasibility of our approach. Bioinformatics analysis of the TssK and TssG proteins over 17 pathogens and corresponding sequence variants (2,482 for TssK and 3,712 for TssG) revealed the high conservation level of the TssK target as well as the preserved triangular hydrophobic core binding motifs, suggesting a broad inhibitory potential. We propose that our peptide could serve as a scaffold to design large-spectrum antivirulence molecules and hence pave the way for the development of anti-T6SS inhibitors.

## MATERIALS AND METHODS

### Data gathering.

TssK, TssG, and TssB EAEC sequences were obtained from the genome of EAEC strain 55989 (Table S1C). Importantly, a single species might have more than one T6SS gene cluster. Using KEGG (76) together with SecReT6 (77) annotations, we selected TssK, TssG, and TssB sequences from the 3 different T6SS gene clusters on the EAEC genome (53, 78, 79). The first gene cluster (EAEC1) belongs to subtype i1, the second (EAEC2) to subtype i4b, and the third (EAEC3) to subtype i2. In the present study, we used EAEC3 as our T6SS model thanks to the available structural data of the TssK-TssG interface (wedge complex cryo-electron microscopy [EM] atomic structures; PDB IDs 6GIY and 6N38). TssK, TssG, and TssB sequences for other pathogenic T6SSs examined were also classified into their corresponding T6SS subtypes following the information retrieved from KEGG and SecreT6 databases (Table S1B and C). Uniref90 (80) was downloaded and converted into a BLAST database using the makeblastdb application (81). We also downloaded all bacterial genomes contained in the SGB collection metagenomic database (82). Using ORFM (83), we identified all possible genes and translated their open reading frames from which putative protein sequences were converted into a BLAST database, using makeblastdb.

### Homologous search.

The search of homologous sequences for TssK, TssG, and TssB was conducted using BLASTp (81) on the Uniref90 (80) and SGB databases (82). We queried the TssK, TssG, and TssB sequences from T6SS EAEC1, EAEC2, and EAEC3 gene clusters, as well as all the gene clusters from a selected list of pathogenic T6SSs (Table S1C). All these sequences, belonging to different gene clusters and pathogens, are referred as to the query sequences here. Homologous sequences whose length was less than 50% or more than 150% of the query sequence length were discarded. All the sequences resulting from the multiple BLASTp searches were merged and clustered by 0.9 sequence identity using cd-hit (84).

### Multiple sequence alignment.

TssK, TssG, and TssB multiple-sequence alignments (MSAs) were generated by multiple rounds of (i) homologous sequence alignment performed by MUSCLE (85) and (ii) removal of outliers by EvalMSA (86). After the last iteration, we manually curated the MSAs using JalView (87).

### Clusters of variants.

Each sequence from the MSA of a given protein was assigned to the most similar query sequence, using blosum62 as a similarity score (88). The assignment allowed us to cluster all the sequences, where each cluster is a set of closely homologous variants of the corresponding query. Clusters of variants were not overlapping, i.e., they did not have any sequence in common. For each cluster, we removed the outlier sequences of the cluster by removing the lowest-scoring 25% of sequences. Similarly, we generated the cluster of variants also for the interacting domains, i.e., TssG foot 1 and 2 and the TssK NTD, by extracting the sequence of the domains from the corresponding MSA.

### Closeness metric, conservation, and similarity level between clusters of variants.

To quantitatively measure the conservation level of a cluster of variants and also the similarity level between different clusters of variants, we defined a closeness metric between two clusters. Given two clusters of aligned sequences, *X* = {*s_i_*} and *Y* = {*s_j_*} with *n* and *m* aligned sequences, respectively, we can compute their closeness as

$$c(X,Y)\;=\;\frac{\text{score}(X,Y)\;-\;\text{randscore}(X,Y)}{\text{idenscore}(X,Y)\;-\;\text{score}(X,Y)}$$

where the score between *X* and *Y* is

$$\text{score}(X,Y)\;=\;\frac{\sum_{i=1}^{n}\sum_{j=1}^{m}\mathrm{sb}62(s_{i},s_{j})}{n^{.}m}$$

and *sb*62 is the blosum62 score between the aligned sequences *s_i_* and *s_j_*. Randscore is the minimum score that one can get between two clusters of sequences:

$$\text{randscore}(X,Y)\;=\;\frac{\sum_{i=1}^{n}\sum_{j=1}^{m}\mathrm{sb}62\left(r(s_{i}),r(s_{j})\right)}{n^{.}m}$$

where the function *r*(*s_i_*) randomizes the sequence *s_i_*, keeping the same length and amino acid composition. Idenscore is the score obtained using identical sequences; thus, it is the highest score one can get:

$$\text{idenscore}(X,Y)\;=\;\frac{\sum_{i=1}^{n}\mathrm{sb}62(s_{i},s_{i})\;+\;\sum_{j=1}^{m}\mathrm{sb}62(s_{j},s_{j})}{n\;+\;m}$$

Note that the closeness *c*(*X*,*Y*) is a normalized quantity, i.e., it ranges between 0 (as far as random clusters) and 1 (as close as identical clusters). Furthermore, the metric is independent of the length of the aligned sequences. This metric computes the similarity level between clusters of sequences when *X* is not equal to *Y*, and it computes the conservation level of a cluster when *X* is equal to *Y*. The conservation level of a cluster of <5 variants is set to 0.

### Data analysis, visualization, and storage.

Residue conservation was computed with JalView (87) and mapped onto the wedge complex structure (PDB ID 6N38) using Chimera (89) (Fig. 2A and B). Residue frequencies (Fig. 2C and D and Fig. S6E) were mapped on the alignment using the WebLogo (90) server. MSA figures (Fig. S8) were generated with JalView. Hierarchical clustering figures (Fig. 6 and Fig. S7) were generated with seaborn (open-source Python package for statistical data visualization) (91). All molecular visualizations were rendered by UCSF Chimera (89) All Python and shell scripts specifically developed for this work are available together with the initial genomic data (TssK, TssB, and TssG sequences) and the resulting MSA (TssK, TssK NTD, TssB, TssG, TssG foot 1, and TssG foot 2) at https://gitlab.pasteur.fr/ifilella/closeness-metric.

### Peptide design.

We started by selecting a six-residue sequence based on the EAEC3 foot 1 sequence (Fig. 2D). We selected the sequence residues of the second variable region (SRPV) together with the third hydrophobic repeat (MG): SRPVMG (positions 238 to 243). Then, we used this sequence to create a cyclic peptide, as follows. The structure of the chosen sequence was cloned twice, applying C3 symmetry about the trimeric TssK axis of symmetry. The three resulting peptide structures were linked through the backbone, introducing N-C bonding restraints (bond, angle, and dihedral restraints introduced by the PATCH command of CHARMM). We included an N-to-C-terminus bonding restraint, resulting in a head-to-tail cyclization of the peptide. Finally, the cyclic peptide was relaxed during a short molecular dynamics (MD) simulation to ensure its stability (Fig. 4).

### Control peptide design.

Two different control peptides were rationally designed: a mutated BCP and a randomized BCP. The mutated BCP was obtained by simply mutating the three Met residues in the 5th position of each BCP repeat to Ala. The randomized peptide was selected from a pool of 13,824 candidates. This pool of candidates was generated by independently permuting the four central residues of each BCP repeat (RPVM), while keeping the Gly and Ser in their original positions to conserve their propensity to form a turn (like the one observed in the foot 1 TssK structure). To design a negative-control peptide, we selected a sequence which we predicted to poorly bind to the trimeric TssK NTD. We ran short MD simulations of all candidates and ranked them according to their total energy after a final minimization. Finally, to avoid any functional redundancy with the original BCP peptide, we selected the peptide that ranked the worst and at the same time did not have any residue at the same position in the BCP sequence (except for the GS residues). The MD simulations of the peptide bound to the TssK NTD were done with CHARMM (92), using the CHARMM22 force field. The initial backbone structure was built from the original TssG foot 1-TssK cryo-EM model, by symmetrizing on the TssG 238-to-243 backbone and the corresponding interacting TssK residues. The modeled TssK NTD sequence was starting from Leu7 and truncated at Phe19. To increase the efficiency of the simulations, we used a distance‐dependent dielectric that mimicked the presence of the solvent. To stabilize the structure of the TssK NTD, we applied an N-terminal acetylation and a C-terminal amidation and restrained the heavy atoms of their terminal residues (residues 7 and 8 and residues 17 to 19) with a harmonic potential centered on their initial positions. The simulations, which ran for 200 ps for each candidate, generated a total of 100 snapshots per sequence. The snapshots were subsequently minimized, and the median of the 100 final total energies was used to rank the peptides (Fig. S8).

### Coevolution analysis.

Residue coevolution identified between TssG and the TssK NTD was detected by combining RaptorX (93) complex contact prediction and Gremlin (94) monomer contact prediction. First, we extracted the concatenated MSA generated by RaptorX when TssK and TssG EAEC sequences were input. Second, we trimmed the TssK NTD and TssG foot 1 from the Raptor MSA and used the resulting concatenated MSA as input for a Gremlin monomer contact prediction search. Finally, we selected the TssK-TssG pairs with a probability above a given threshold and a distance between their side chains below 8 Å on the atomic structure of the wedge structure (PDB ID 6N38).

### Peptide synthesis.

The biomimetic cyclic peptide was synthesized by GenicBio, Ltd.

### Plasmid construction.

PCRs were performed using Q5 high-fidelity DNA polymerase (New England Biolabs). Restriction enzymes and T4 DNA polymerase were purchased from New England Biolabs and used according to the manufacturer’s instructions. Custom oligonucleotides were synthetized by Sigma-Aldrich and are listed in Table S1A. EAEC 17-2, Acinetobacter baumannii 17978, and P. aeruginosa PAO1 chromosomal DNA were used as templates for PCRs. E. coli DH5α was used for cloning procedures. Apart from the pKO3-*tssG*-Cterm vector, plasmids were engineered by a two-fragments sequence- and ligation-independent cloning (SLIC) strategy (95, 96). Briefly, each DNA fragments were amplified by PCR using two pairs of oligonucleotides (FWD1/REV1 and FWD2/REV2). PCR products were digested (DpnI), cleaned (Macherey-Nagel PCR cleaning kit), and mixed together with the T4 DNA polymerase and its buffer (NEBuffer r2.1) at room temperature. The reaction was stopped after 2 min 45 s, and the mixture was put on ice. The annealing product was transformed into competent E. coli DH5α, and recombinant strains were selected on the appropriate antibiotic. Substitutions in pTRC-99A-TssK^H^ and pRSF-TssK^S^ were introduced by site-directed mutagenesis using complementary pairs of oligonucleotides. All constructs were verified by DNA sequencing.

### Protein production and purification.

Plasmids expressing the gene combinations of interest were cotransformed into E. coli BL21(DE3), as described in “Plasmid construction” above. Cells were grown at 37°C in lysogeny broth (LB) to an *A*_600_ of ∼0.7, and gene expression was induced by the addition of 1 mM IPTG (isopropyl-β-d-thiogalactopyranoside) for 16 h at 16°C. Cell pellets were suspended in 50 mM Tris-HCl (pH 8.0), 150 mM NaCl, 1 mM EDTA, 10 mM MgCl_2_ supplemented with 10 μg/ml of DNase I, 100 μg/ml of lysozyme, and EDTA-free protease inhibitor (Roche) to an *A*_600_ of ∼125. Cells were broken using an Emulsiflex-C5 instrument (Avestin) and clarified by centrifugation for 30 min at 20,000 × *g*. The supernatant was loaded onto a 5-ml StrepTrap HP (GE Healthcare) column on an Äkta Pure system (GE Healthcare) equilibrated in affinity buffer (50 mM Tris-HCl [pH 8.0], 150 mM NaCl). The column was then washed using the affinity buffer, and the proteins were eluted in the same buffer supplemented with 2.5 mM desthiobiotin (IBA Technology). The lysate, flowthrough, wash, and elution fractions were collected, suspended in Laemmli loading buffer supplemented with 1 mM 2-mercaptoethanol, and heated for 10 min at 96°C prior to analyses by SDS-PAGE and immunoblotting. For the study of the interaction between TssK and TssG variants, copurification was carried out by pulling down Strep-tagged TssK and evaluating the presence of copurified TssG by Western blotting.

### SDS-PAGE, protein transfer, immunostaining, and antibodies.

SDS-PAGE was performed on Bio-Rad Mini-Protean systems using standard protocols with homemade 12.5% polyacrylamide gels. For immunostaining, proteins were transferred onto 0.2-μm nitrocellulose membranes (Amersham Protran) with a Mini-Trans Blot cell (Bio-Rad). The membrane was then saturated in 5% milk and probed with primary antibodies. Mouse secondary antibody coupled to alkaline phosphatase was added and developed in alkaline buffer in the presence of 5-bromo-4-chloro-3-indolylphosphate and nitroblue tetrazolium. The antihemagglutinin (HA) (HA-7 clone; Sigma-Aldrich), anti-Flag (M2 clone; Sigma-Aldrich), anti-StrepII (Sigma-Aldrich), anti-5His (Sigma-Aldrich) monoclonal antibodies, and mouse secondary antibodies (Millipore) were purchased as indicated.

### Fluorescence microscopy.

Fluorescence microscopy experiments were performed as described elsewhere (97, 98). Briefly, cells were grown overnight in LB and diluted to an *A*_600_ of ∼0.04 in SIM (sulfur, indole, and motility) medium. Exponentially growing cells (*A*_600_ ∼ 0.8 to 1) were harvested, washed in phosphate-buffered saline (PBS) buffer, resuspended in PBS to an *A*_600_ of ∼50, spotted on a 2% agarose pad, and covered with a coverslip. Fluorescence and phase-contrast micrographs were captured using an AxioImager M2 microscope (Zeiss) equipped with an OrcaR2 digital camera (Hamamatsu). Fluorescence images were acquired with a minimal exposure time to reduce bleaching and phototoxicity effects, typically 500 ms for TssK-sfGFP. Noise and background were reduced using the Subtract Background (20 pixels, rolling ball) and Band plugins of ImageJ (National Institutes of Health). The sfGFP foci were automatically detected using the MicrobeJ plugin (https://www.microbej.com/). Box plots representing the number of detected foci for each strain were made using the Web tool BoxPlotR (http://shiny.chemgrid.org/boxplotr/). Microscopy analyses were performed at least three times, each in technical triplicate, and results of a representative experiment are shown.

### Strain construction.

Deletions of the TssG foot 1 and foot 2 domains were engineered at the native locus on the chromosome by allelic replacement using the pKO3 suicide vector (99) in the enteroaggregative E. coli 17-2 and E. coli 17-2 TssK-sfGFP strains. Briefly, the E. coli strains were transformed with a pKO3 plasmid in which a portion of TssG harboring the domain deletion has been cloned (see below). Insertion of the plasmid into the chromosome was selected on chloramphenicol plates at 42°C. The removal of the plasmid fragment was then selected on 5% sucrose plates without antibiotic, and mutations were screened by PCR and confirmed by DNA sequencing (Eurofins, MWG).

### Native polyacrylamide gel electrophoresis.

Plasmids expressing the gene combinations of interest (pCDF-TssK^H^, TssK^H^-^S^TssF-TssG^Δfoot1-F^-^HA^TssE, and TssK^H^-^S^TssF-TssG^Δfoot2-F^-^HA^TssE) were cotransformed into E. coli BL21(DE3) as described in “Plasmid construction” above. Cells were grown at 37°C in LB to an *A*_600_ of ∼0.7, and expression of the target genes was induced by addition of 1 mM IPTG for 16 h at 16°C. Cell pellets were suspended in 50 mM Tris-HCl (pH 8.0), 150 mM NaCl, 1 mM EDTA, 10 mM MgCl_2_ supplemented with 100 mg/ml of DNase I, 100 mg/ml of lysozyme, and EDTA-free protease inhibitor (Roche) to an *A*_600_ of ∼125. Cells were broken using an Emulsiflex-C5 instrument (Avestin) and clarified by centrifugation for 30 min at 20,000 × *g*. After clarification, lysates were loaded on a native 4 to 16% gel (Mini-Protean TGX; Bio-Rad). After migration, proteins and protein complexes were transferred onto a nitrocellulose membrane and immunoblotted as described above.

### Interbacterial competition assay.

The antibacterial growth competition assay was performed as previously described (100). Wild-type E. coli K-12 strain W3110 bearing the pUA66-rrnB plasmid (conferring kanamycin resistance and constitutive GFP fluorescence, with the *gfp* gene under the control of the ribosomal *rrnB* promoter) (101) was used as the recipient. Attacker and recipient cells were grown for 16 h in LB medium, diluted in SIM medium to allow maximal expression of the *sci-1* gene cluster. Once the culture reached an *A*_600_ of ∼0.8, cells were harvested and normalized, and drops of the mixture were spotted in triplicate onto a prewarmed dry SIM agar plate with or without the addition of 0.5 mg ml^−1^ arabinose. After incubation for 4 h at 37°C, the bacterial spots were resuspended in LB, and bacterial suspensions were normalized to an *A*_600_ of 0.5. For the enumeration of viable prey cells, bacterial suspensions were serially diluted and spotted onto kanamycin LB plates. The assays were performed from at least three independent cultures, with technical triplicates, and results of a representative technical triplicate are shown.

### Biolayer interferometry experiment.

Streptavidin-tagged TssK was purified as described above. The TssK trimer was further polished by size exclusion chromatography using a Superose 6 10/300 GL (GE Healthcare) in 50 mM HEPES (pH 7.0), 150 mM NaCl at 0.25 ml/min. For biolayer interferometry (BLI) experiments, we used high-precision Strep (SAX) biosensors from Forté Bio (number 18-5117) on the BLItz machine (Forté Bio). The trimeric ligand TssK was biotinylated using equimolar *N*-hydroxysuccinimide (NHS)–polyethylene glycol 4 (PEG4)–biotin (Pierce EZ-Link; 21330) in 50 mM HEPES (pH 7.0), NaCl 150 mM for 1 h at room temperature before buffer exchange in CentriPure P2 (Generon; IRL) columns against 50 mM HEPES (pH 7.0), NaCl 150 mM to remove the excess biotin. BL21(DE3) cells were used to express the interacting analytes TssG-TssF-TssE. In addition, cells containing an empty plasmid were used as a control. Cells were broken as described above, and the clarified supernatants after centrifugation for 30 min at 20,000 × *g* contained the control and TssFGE lysate. Streptavidin biosensors were hydrated for 10 min in HEPES buffer before binding of the biotinylated TssK. Biotinylated TssK was loaded at 1.2 μM onto the SAX biosensors for 120 s, and then the excess TssK was dissociated in HEPES buffer for 120 s. For the remaining kinetic assays, we used kinetic buffer (PBS [pH 7.3] plus 0.02% Tween 20, 0.1% bovine serum albumin [BSA], 0.05% sodium azide) (Forté Bio number 18-5032) with orbital shaking at 2,200 rpm. Kinetic buffer (KB) was used to dilute the lysates and the peptide to decrease nonspecific signals. The unbound Strep sites were blocked with 2 μM control lysate for 120 s and washed with KB for 120 s. The kinetic assays were performed using three steps with different times: baseline, 30 s; association, 120 s; and dissociation, 120 s. All the following assays were performed in triplicate. Four independent assays were performed to demonstrate the specific binding of TssK with its natural partners TssF-TssG-TssE and the effect of the wild type biomimetic peptide to block or hinder the specific binding of the analytes. In the first assay, we added 47 μM lysate expressing the analytes TssG, TssF, and TssE to the bound TssK. In the second assay, we added 1 mM wild-type (WT) BCP to the bound TssK before adding 47 μM TssF-TssG-TssE. In the third assay, to validate the specificity of the WT BCP we added 1 mM mutant BCP that lacks the binding residues to the bound TssK before adding 47 μM TssF-TssG-TssE. Last, to redemonstrate the specificity of the BCP-WT, we added 1 mM randomized BCP that contains the binding residues but out of order to the bound TssK before adding 47 μM TssF-TssG-TssE.

### ITC.

Isothermal titration calorimetry (ITC) was performed to demonstrate the specific interaction of TssK with WT BCP and the lack of interaction with TssK L14A. The working buffer for both proteins and peptide was 50 mM HEPES (pH 7.0), 150 mM NaCl to avoid buffer mismatch. The experiments were performed at 25°C using the MicroCal PEAQ-ITC instrument (Malvern Panalyticals, Malvern, UK) in duplicate with 19 injections, first with an initial injection of 0.4 μl followed by 18 injections of 2 μl. Two assays were performed. In the first, 25 μM WT TssK ligand was in the cell and 1 mM WT BCP analyte was in the syringe. In the second, 25 μM TssK L14A ligand was in the cell and 1 mM WT BCP analyte was in the syringe. The reaction was performed with a constant stirring speed of 750 rpm; each injection lasted for 4 s with a 150-s space between injections. A constant heat control (offset) was removed from the raw data to account for heat dilution before integration. The data were fitted using the One Set of Sites model in the PEAQ-ITC analysis software.

### TssK focus *in vivo* inhibition.

Fluorescence microscopy experiments were performed as previously described (97, 98). An overnight culture in LB of EAEC strain with a chromosomal GFP fusion to TssK was diluted 1/200 in SIM medium and grown at 37°C until the culture reached an *A*_600_ of ∼0.8. Ten optical density units (ODU) was pelleted for 10 min at 3,500 × *g*, resuspended at 1 ODU/ml in the same SIM medium supplemented with 0.8 M NaCl, and equilibrated for 1 h at room temperature. Cells were pelleted for 10 min at 3,500 × *g*, resuspended in 100 μl of classical SIM without NaCl supplemented with 1 mM peptide of buffer, and incubated for 20 min at room temperature. Cells were diluted in classical SIM medium at 0.4 ODU/ml and incubated at 37°C 160 rpm until the absorbance again reached ∼0.8. Ten ODU was harvested and resuspended in fresh SIM medium. Cell mixtures were spotted on a thin pad of SIM medium supplemented with 2% agarose, covered with a coverslip, and incubated for 20 to 30 min at room temperature before microscopy acquisition. Fluorescence microscopy was performed with a Nikon Eclipse Ti2 microscope equipped with a 100× objective (numerical aperture, 1.45), an Orca-Fusion digital camera (Hamamatsu), and a perfect focus system (PFS) to automatically maintain focus so that the point of interest within a specimen is kept in sharp focus at all times despite mechanical or thermal perturbations. All fluorescence images were acquired in Hilo mode using an Ilas2 TIRF module (Gataca Systems). Exposure times were typically 100 ms for phase contrast and 100 ms using the GFP channel. For image treatment, noise and background were reduced by filtering large structures down to 40 pixels in the FFT Bandpass Filter function of ImageJ (102). The GFP foci were automatically detected using the MicrobeJ plugin (103).

### NMR experiments.

All NMR experiments were recorded at 300 K on a Bruker Avance-II 600 MHz spectrometer equipped with a cryoprobe at the IMM (Institut de Microbiologie de la Mediterranée) NMR platform. The 450-μl peptide sample tube was prepared at 1.8 mM in KPO_4_ buffer (50 mM KPO_4_ [pH 6.9], 150 mM NaCl) complemented with 30 μl D_2_O. 1D ^1^H-^1^H-TOCSY, ^1^H-NOESY, and ^1^H-^15^N HSQC spectra (F2 = 2048; F1 = 128; NS = 384) (NS is the number of scans [accumulation] for the experiment; F2 is the sweep width; F1 is the Fourier number that defines the 2D spectrum dimensions and its digital resolution) were recorded using default pulse sequences as provided by the manufacturer. For the peptide-TssK interaction experiments, 200 μl of a 410 μM stock of TssK was added to the same sample tube (1.2 mM peptide and 140 μM TssK [final concentration]), and all NMR spectra were recorded with the addition of longer ^1^H-^15^N HSQC spectrometry (NS = 1,024). All spectra were transformed and the figures were generated using Bruker Topspin 4.0.9.

10.1128/mBio.01348-21.9 FIG S9 Synthesis and quality data of the BCP. (A) High-performance liquid chromatography (HPLC) result and table summarizing the synthesis parameters. (B) LC-mass spectrometry (MS) measurement of the BCP. Download FIG S9, TIF file, 1.7 MB. Copyright © 2021 Cherrak et al. 2021 Cherrak et al. https://creativecommons.org/licenses/by/4.0/ This content is distributed under the terms of the Creative Commons Attribution 4.0 International license.

## References

[1] Silver LL. 2011. Challenges of antibacterial discovery. Clin Microbiol Rev 24:71–109. doi:10.1128/CMR.00030-10. 21233508 PMC3021209

[2] Boucher HW, Talbot GH, Bradley JS, Edwards JE, Gilbert D, Rice LB, Scheld M, Spellberg B, Bartlett J. 2009. Bad bugs, no drugs: no ESKAPE! An update from the Infectious Diseases Society of America. Clin Infect Dis 48:1–12. doi:10.1086/595011. 19035777

[3] Cassini A, Högberg LD, Plachouras D, Quattrocchi A, Hoxha A, Simonsen GS, Colomb-Cotinat M, Kretzschmar ME, Devleesschauwer B, Cecchini M, Ouakrim DA, Oliveira TC, Struelens MJ, Suetens C, Monnet DL, Strauss R, Mertens K, Struyf T, Catry B, Latour K, Ivanov IN, Dobreva EG, Tambic Andraševic A, Soprek S, Budimir A, Paphitou N, Žemlicková H, Schytte Olsen S, Wolff Sönksen U, Märtin P, Ivanova M, Lyytikäinen O, Jalava J, Coignard B, Eckmanns T, Abu Sin M, Haller S, Daikos GL, Gikas A, Tsiodras S, Kontopidou F, Tóth Á, Hajdu Á, Guólaugsson Ó, Kristinsson KG, Murchan S, Burns K, Pezzotti P, Gagliotti C, Dumpis U, Burden of AMR Collaborative Group, et al. 2019. Attributable deaths and disability-adjusted life-years caused by infections with antibiotic-resistant bacteria in the EU and the European Economic Area in 2015: a population-level modelling analysis. Lancet Infect Dis 19:56–66. doi:10.1016/S1473-3099(18)30605-4. 30409683 PMC6300481

[4] Casadevall A, Pirofski LA. 1999. Host-pathogen interactions: redefining the basic concepts of virulence and pathogenicity. Infect Immun 67:3703–3713. doi:10.1128/IAI.67.8.3703-3713.1999. 10417127 PMC96643

[5] Allen RC, Popat R, Diggle SP, Brown SP. 2014. Targeting virulence: can we make evolution-proof drugs? Nat Rev Microbiol 12:300–308. doi:10.1038/nrmicro3232. 24625893

[6] Dickey SW, Cheung GYC, Otto M. 2017. Different drugs for bad bugs: antivirulence strategies in the age of antibiotic resistance. Nat Rev Drug Discov 16:457–471. doi:10.1038/nrd.2017.23. 28337021 PMC11849574

[7] Calvert MB, Jumde VR, Titz A. 2018. Pathoblockers or antivirulence drugs as a new option for the treatment of bacterial infections. Beilstein J Org Chem 14:2607–2617. doi:10.3762/bjoc.14.239. 30410623 PMC6204809

[8] Green ER, Mecsas J. 2016. Bacterial secretion systems: an overview. Microbiol Spectr 4:VMBF-0012-2015. doi:10.1128/microbiolspec.VMBF-0012-2015. PMC4804464 26999395

[9] Costa TR, Felisberto-Rodrigues C, Meir A, Prevost MS, Redzej A, Trokter M, Waksman G. 2015. Secretion systems in Gram-negative bacteria: structural and mechanistic insights. Nat Rev Microbiol 13:343–359. doi:10.1038/nrmicro3456. 25978706

[10] Pukatzki S, Ma AT, Sturtevant D, Krastins B, Sarracino D, Nelson WC, Heidelberg JF, Mekalanos JJ. 2006. Identification of a conserved bacterial protein secretion system in *Vibrio cholerae* using the Dictyostelium host model system. Proc Natl Acad Sci U S A 103:1528–1533. doi:10.1073/pnas.0510322103. 16432199 PMC1345711

[11] Mougous JD, Cuff ME, Raunser S, Shen A, Zhou M, Gifford CA, Goodman AL, Joachimiak G, Ordoñez CL, Lory S, Walz T, Joachimiak A, Mekalanos JJ. 2006. A virulence locus of *Pseudomonas aeruginosa* encodes a protein secretion apparatus. Science 312:1526–1530. doi:10.1126/science.1128393. 16763151 PMC2800167

[12] Bingle LE, Bailey CM, Pallen MJ. 2008. Type VI secretion: a beginner's guide. Curr Opin Microbiol 11:3–8. doi:10.1016/j.mib.2008.01.006. 18289922

[13] Boyer F, Fichant G, Berthod J, Vandenbrouck Y, Attree I, 10. 2009. Dissecting the bacterial type VI secretion system by a genome wide in silico analysis: what can be learned from available microbial genomic resources? BMC Genomics 10:104. doi:10.1186/1471-2164-10-104. 19284603 PMC2660368

[14] Sana TG, Baumann C, Merdes A, Soscia C, Rattei T, Hachani A, Jones C, Bennett KL, Filloux A, Superti-Furga G, Voulhoux R, Bleves S. 2015. Internalization of *Pseudomonas aeruginosa* strain PAO1 into epithelial cells is promoted by interaction of a T6SS effector with the microtubule network. mBio 6:e00712-15. doi:10.1128/mBio.00712-15. 26037124 PMC4453011

[15] Suarez G, Sierra JC, Erova TE, Sha J, Horneman AJ, Chopra AK. 2010. A type VI secretion system effector protein, VgrG1, from *Aeromonas hydrophila* that induces host cell toxicity by ADP ribosylation of actin. J Bacteriol 192:155–168. doi:10.1128/JB.01260-09. 19880608 PMC2798274

[16] Pukatzki S, Ma AT, Revel AT, Sturtevant D, Mekalanos JJ. 2007. Type VI secretion system translocates a phage tail spike-like protein into target cells where it cross-links actin. Proc Natl Acad Sci U S A 104:15508–15513. doi:10.1073/pnas.0706532104. 17873062 PMC2000545

[17] Ma AT, Mekalanos JJ. 2010. In vivo actin cross-linking induced by *Vibrio cholerae* type VI secretion system is associated with intestinal inflammation. Proc Natl Acad Sci U S A 107:4365–4370. doi:10.1073/pnas.0915156107. 20150509 PMC2840160

[18] Suarez G, Sierra JC, Kirtley ML, Chopra AK. 2010. Role of Hcp, a type 6 secretion system effector, of *Aeromonas hydrophila* in modulating activation of host immune cells. Microbiology (Reading) 156:3678–3688. doi:10.1099/mic.0.041277-0. 20798163 PMC3068704

[19] Barker JR, Chong A, Wehrly TD, Yu JJ, Rodriguez SA, Liu J, Celli J, Arulanandam BP, Klose KE. 2009. The *Francisella tularensis* pathogenicity island encodes a secretion system that is required for phagosome escape and virulence. Mol Microbiol 74:1459–1470. doi:10.1111/j.1365-2958.2009.06947.x. 20054881 PMC2814410

[20] Burtnick MN, DeShazer D, Nair V, Gherardini FC, Brett PJ. 2010. *Burkholderia mallei* cluster 1 type VI secretion mutants exhibit growth and actin polymerization defects in RAW 264.7 murine macrophages. Infect Immun 78:88–99. doi:10.1128/IAI.00985-09. 19884331 PMC2798217

[21] Wang T, Si M, Song Y, Zhu W, Gao F, Wang Y, Zhang L, Zhang W, Wei G, Luo ZQ, Shen X. 2015. Type VI secretion system transports Zn2+ to combat multiple stresses and host immunity. PLoS Pathog 11:e1005020. doi:10.1371/journal.ppat.1005020. 26134274 PMC4489752

[22] Chen H, Yang D, Han F, Tan J, Zhang L, Xiao J, Zhang Y, Liu Q. 2017. The bacterial T6SS effector EvpP prevents NLRP3 inflammasome activation by inhibiting the Ca2+-dependent MAPK-Jnk pathway. Cell Host Microbe 21:47–58. doi:10.1016/j.chom.2016.12.004. 28081443

[23] Repizo GD, Gagné S, Foucault-Grunenwald ML, Borges V, Charpentier X, Limansky AS, Gomes JP, Viale AM, Salcedo SP. 2015. Differential role of the T6SS in *Acinetobacter baumannii* virulence. PLoS One 10:e0138265. doi:10.1371/journal.pone.0138265. 26401654 PMC4581634

[24] Sana TG, Flaugnatti N, Lugo KA, Lam LH, Jacobson A, Baylot V, Durand E, Journet L, Cascales E, Monack DM. 2016. *Salmonella* Typhimurium utilizes a T6SS-mediated antibacterial weapon to establish in the host gut. Proc Natl Acad Sci U S A 113:E5044–E5051. doi:10.1073/pnas.1608858113. 27503894 PMC5003274

[25] Anderson MC, Vonaesch P, Saffarian A, Marteyn BS, Sansonetti PJ. 2017. *Shigella sonnei* encodes a functional T6SS used for interbacterial competition and niche occupancy. Cell Host Microbe 21:769–776.e3. doi:10.1016/j.chom.2017.05.004. 28618272

[26] Fast D, Kostiuk B, Foley E, Pukatzki S. 2018. Commensal pathogen competition impacts host viability. Proc Natl Acad Sci U S A 115:7099–7104. doi:10.1073/pnas.1802165115. 29915049 PMC6142279

[27] Zhao W, Caro F, Robins W, Mekalanos JJ. 2018. Antagonism toward the intestinal microbiota and its effect on *Vibrio cholerae* virulence. Science 359:210–213. doi:10.1126/science.aap8775. 29326272 PMC8010019

[28] Sun K, Bröms J, Lavander M, Gurram BK, Enquist PA, Andersson CD, Elofsson M, Sjöstedt A. 2014. Screening for inhibition of *Vibrio cholerae* VipA-VipB interaction identifies small-molecule compounds active against type VI secretion. Antimicrob Agents Chemother 58:4123–4130. doi:10.1128/AAC.02819-13. 24798289 PMC4068513

[29] Leiman PG, Shneider MM. 2012. Contractile tail machines of bacteriophages. Adv Exp Med Biol 726:93–114. doi:10.1007/978-1-4614-0980-9_5. 22297511

[30] Basler M. 2015. Type VI secretion system: secretion by a contractile nanomachine. Philos Trans R Soc B 370:20150021. doi:10.1098/rstb.2015.0021. PMC4632598 26370934

[31] Ge P, Scholl D, Leiman PG, Yu X, Miller JF, Zhou ZH. 2015. Atomic structures of a bactericidal contractile nanotube in its pre- and postcontraction states. Nat Struct Mol Biol 22:377–382. doi:10.1038/nsmb.2995. 25822993 PMC4445970

[32] Böck D, Medeiros JM, Tsao HF, Penz T, Weiss GL, Aistleitner K, Horn M, Pilhofer M. 2017. In situ architecture, function, and evolution of a contractile injection system. Science 357:713–717. doi:10.1126/science.aan7904. 28818949 PMC6485382

[33] Basler M, Pilhofer M, Henderson GP, Jensen GJ, Mekalanos JJ. 2012. Type VI secretion requires a dynamic contractile phage tail-like structure. Nature 483:182–186. doi:10.1038/nature10846. 22367545 PMC3527127

[34] Ballister ER, Lai AH, Zuckermann RN, Cheng Y, Mougous JD. 2008. In vitro self-assembly of tailorable nanotubes from a simple protein building block. Proc Natl Acad Sci U S A 105:3733–3738. doi:10.1073/pnas.0712247105. 18310321 PMC2268831

[35] Leiman PG, Basler M, Ramagopal UA, Bonanno JB, Sauder JM, Pukatzki S, Burley SK, Almo SC, Mekalanos JJ. 2009. Type VI secretion apparatus and phage tail-associated protein complexes share a common evolutionary origin. Proc Natl Acad Sci U S A 106:4154–4159. doi:10.1073/pnas.0813360106. 19251641 PMC2657435

[36] Brunet YR, Hénin J, Celia H, Cascales E. 2014. Type VI secretion and bacteriophage tail tubes share a common assembly pathway. EMBO Rep 15:315–321. doi:10.1002/embr.201337936. 24488256 PMC3989698

[37] Brackmann M, Wang J, Basler M. 2018. Type VI secretion system sheath inter-subunit interactions modulate its contraction. EMBO Rep 19:225–233. doi:10.15252/embr.201744416. 29222345 PMC5797969

[38] Aschtgen MS, Gavioli M, Dessen A, Lloubès R, Cascales E. 2010. The SciZ protein anchors the enteroaggregative *Escherichia coli* type VI secretion system to the cell wall. Mol Microbiol 75:886–899. doi:10.1111/j.1365-2958.2009.07028.x. 20487285

[39] Durand E, Nguyen VS, Zoued A, Logger L, Péhau-Arnaudet G, Aschtgen MS, Spinelli S, Desmyter A, Bardiaux B, Dujeancourt A, Roussel A, Cambillau C, Cascales E, Fronzes R. 2015. Biogenesis and structure of a type VI secretion membrane core complex. Nature 523:555–560. doi:10.1038/nature14667. 26200339

[40] Rapisarda C, Cherrak Y, Kooger R, Schmidt V, Pellarin R, Logger L, Cascales E, Pilhofer M, Durand E, Fronzes R. 2019. In situ and high-resolution cryo-EM structure of a bacterial type VI secretion system membrane complex. EMBO J 38:e100886. doi:10.15252/embj.2018100886. 30877094 PMC6517824

[41] Russell AB, Peterson SB, Mougous JD. 2014. Type VI secretion system effectors: poisons with a purpose. Nat Rev Microbiol 12:137–148. doi:10.1038/nrmicro3185. 24384601 PMC4256078

[42] Durand E, Cambillau C, Cascales E, Journet L. 2014. VgrG, Tae, Tle, and beyond: the versatile arsenal of type VI secretion effectors. Trends Microbiol 22:498–507. doi:10.1016/j.tim.2014.06.004. 25042941

[43] Alcoforado Diniz J, Liu YC, Coulthurst SJ. 2015. Molecular weaponry: diverse effectors delivered by the type VI secretion system. Cell Microbiol 17:1742–1751. doi:10.1111/cmi.12532. 26432982 PMC4832377

[44] Hachani A, Wood TE, Filloux A. 2016. Type VI secretion and anti-host effectors. Curr Opin Microbiol 29:81–93. doi:10.1016/j.mib.2015.11.006. 26722980

[45] Brunet YR, Zoued A, Boyer F, Douzi B, Cascales E. 2015. The type VI secretion TssEFGK-VgrG phage-like baseplate is recruited to the TssJLM membrane complex via multiple contacts and serves as assembly platform for tail tube/sheath polymerization. PLoS Genet 11:e1005545. doi:10.1371/journal.pgen.1005545. 26460929 PMC4604203

[46] Nguyen VS, Logger L, Spinelli S, Legrand P, Huyen Pham TT, Nhung Trinh TT, Cherrak Y, Zoued A, Desmyter A, Durand E, Roussel A, Kellenberger C, Cascales E, Cambillau C. 2017. Type VI secretion TssK baseplate protein exhibits structural similarity with phage receptor-binding proteins and evolved to bind the membrane complex. Nat Microbiol 2:17103. doi:10.1038/nmicrobiol.2017.103. 28650463

[47] Vettiger A, Winter J, Lin L, Basler M. 2017. The type VI secretion system sheath assembles at the end distal from the membrane anchor. Nat Commun 8:16088. doi:10.1038/ncomms16088. 28703218 PMC5511345

[48] English G, Byron O, Cianfanelli FR, Prescott AR, Coulthurst SJ. 2014. Biochemical analysis of TssK, a core component of the bacterial type VI secretion system, reveals distinct oligomeric states of TssK and identifies a TssK-TssFG subcomplex. Biochem J 461:291–304. doi:10.1042/BJ20131426. 24779861 PMC4072051

[49] Taylor NM, Prokhorov NS, Guerrero-Ferreira RC, Shneider MM, Browning C, Goldie KN, Stahlberg H, Leiman PG. 2016. Structure of the T4 baseplate and its function in triggering sheath contraction. Nature 533:346–352. doi:10.1038/nature17971. 27193680

[50] Cherrak Y, Rapisarda C, Pellarin R, Bouvier G, Bardiaux B, Allain F, Malosse C, Rey M, Chamot-Rooke J, Cascales E, Fronzes R, Durand E. 2018. Biogenesis and structure of a type VI secretion baseplate. Nat Microbiol 3:1404–1416. doi:10.1038/s41564-018-0260-1. 30323254

[51] Park YJ, Lacourse KD, Cambillau C, DiMaio F, Mougous JD, Veesler D. 2018. Structure of the type VI secretion system TssK-TssF-TssG baseplate subcomplex revealed by cryo-electron microscopy. Nat Commun 9:5385. doi:10.1038/s41467-018-07796-5. 30568167 PMC6300606

[52] Kritzer JA, Lear JD, Hodsdon ME, Schepartz A. 2004. Helical beta-peptide inhibitors of the p53-hDM2 interaction. J Am Chem Soc 126:9468–9469. doi:10.1021/ja031625a. 15291512

[53] Russell AB, Wexler AG, Harding BN, Whitney JC, Bohn AJ, Goo YA, Tran BQ, Barry NA, Zheng H, Peterson SB, Chou S, Gonen T, Goodlett DR, Goodman AL, Mougous JD. 2014. A type VI secretion-related pathway in Bacteroidetes mediates interbacterial antagonism. Cell Host Microbe 16:227–236. doi:10.1016/j.chom.2014.07.007. 25070807 PMC4136423

[54] Sana TG, Berni B, Bleves S. 2016. The T6SSs of *Pseudomonas aeruginosa* strain PAO1 and their effectors: beyond bacterial-cell targeting. Front Cell Infect Microbiol 6:61. doi:10.3389/fcimb.2016.00061. 27376031 PMC4899435

[55] Chen F, Zhang W, Schwarz S, Zhu Y, Li R, Hua X, Liu S. 2019. Genetic characterization of an MDR/virulence genomic element carrying two T6SS gene clusters in a clinical *Klebsiella pneumoniae* isolate of swine origin. J Antimicrob Chemother 74:1539–1544. doi:10.1093/jac/dkz093. 30903161

[56] Lewis JM, Deveson Lucas D, Harper M, Boyce JD. 2019. Systematic identification and analysis of *Acinetobacter baumannii* type VI secretion system effector and immunity components. Front Microbiol 10:2440. doi:10.3389/fmicb.2019.02440. 31736890 PMC6833914

[57] Navarro-Garcia F, Ruiz-Perez F, Cataldi Á, Larzábal M. 2019. Type VI secretion system in pathogenic *Escherichia coli*: structure, role in virulence, and acquisition. Front Microbiol 10:1965. doi:10.3389/fmicb.2019.01965. 31543869 PMC6730261

[58] Bulterys PL, Toesca IJ, Norris MH, Maloy JP, Fitz-Gibbon ST, France B, Toffig B, Morselli M, Somprasong N, Pellegrini M, Schweizer HP, Tuanyok A, Damoiseaux R, French CT, Miller JF. 2019. An in situ high-throughput screen identifies inhibitors of intracellular *Burkholderia pseudomallei* with therapeutic efficacy. Proc Natl Acad Sci U S A 116:18597–18606. doi:10.1073/pnas.1906388116. 31439817 PMC6744847

[59] Gao X, Mu Z, Qin B, Sun Y, Cui S. 2017. Structure-based prototype peptides targeting the *Pseudomonas aeruginosa* type VI secretion system effector as a novel antibacterial strategy. Front Cell Infect Microbiol 7:411. doi:10.3389/fcimb.2017.00411. 28979890 PMC5611513

[60] Nguyen VS, Logger L, Spinelli S, Desmyter A, Le TT, Kellenberger C, Douzi B, Durand E, Roussel A, Cascales E, Cambillau C. 2015. Inhibition of type VI secretion by an anti-TssM llama nanobody. PLoS One 10:e0122187. doi:10.1371/journal.pone.0122187. 25811612 PMC4374921

[61] Henchey LK, Jochim AL, Arora PS. 2008. Contemporary strategies for the stabilization of peptides in the alpha-helical conformation. Curr Opin Chem Biol 12:692–697. doi:10.1016/j.cbpa.2008.08.019. 18793750 PMC2650020

[62] Siegert TR, Bird MJ, Makwana KM, Kritzer JA. 2016. Analysis of loops that mediate protein–protein interactions and translation into submicromolar inhibitors. J Am Chem Soc 138:12876–12884. doi:10.1021/jacs.6b05656. 27611902

[63] Voter AF, Keck JL. 2018. Development of protein–protein interaction inhibitors for the treatment of infectious diseases. Adv Protein Chem Struct Biol 111:197–222. doi:10.1016/bs.apcsb.2017.07.005. 29459032 PMC6201748

[64] Heinis C, Rutherford T, Freund S, Winter G. 2009. Phage-encoded combinatorial chemical libraries based on bicyclic peptides. Nat Chem Biol 5:502–507. doi:10.1038/nchembio.184. 19483697

[65] Schlippe YV, Hartman MC, Josephson K, Szostak JW. 2012. In vitro selection of highly modified cyclic peptides that act as tight binding inhibitors. J Am Chem Soc 134:10469–10477. doi:10.1021/ja301017y. 22428867 PMC3384292

[66] Hayashi Y, Morimoto J, Suga H. 2012. In vitro selection of anti-Akt2 thioether-macrocyclic peptides leading to isoform-selective inhibitors. ACS Chem Biol Mar 16:607–613. doi:10.1021/cb200388k. 22273180

[67] Hecht AL, Casterline BW, Earley ZM, Goo YA, Goodlett DR, Bubeck Wardenburg J. 2016. Strain competition restricts colonization of an enteric pathogen and prevents colitis. EMBO Rep 17:1281–1291. doi:10.15252/embr.201642282. 27432285 PMC5007561

[68] Wexler AG, Bao Y, Whitney JC, Bobay LM, Xavier JB, Schofield WB, Barry NA, Russell AB, Tran BQ, Goo YA, Goodlett DR, Ochman H, Mougous JD, Goodman AL. 2016. Human symbionts inject and neutralize antibacterial toxins to persist in the gut. Proc Natl Acad Sci U S A 113:3639–3644. doi:10.1073/pnas.1525637113. 26957597 PMC4822603

[69] Coyne MJ, Comstock LE. 2019. Type VI secretion systems and the gut microbiota. Microbiol Spectr 7:PSIB-0009-2018. doi:10.1128/microbiolspec.PSIB-0009-2018. PMC6404974 30825301

[70] Valguarnera E, Wardenburg JB. 2020. Good gone bad: one toxin away from disease for *Bacteroides fragilis*. J Mol Biol 432:765–785. doi:10.1016/j.jmb.2019.12.003. 31857085

[71] Yekani M, Baghi HB, Naghili B, Vahed SZ, Sóki J, Memar MY. 2020. To resist and persist: important factors in the pathogenesis of *Bacteroides fragilis*. Microb Pathog 149:104506. doi:10.1016/j.micpath.2020.104506. 32950639

[72] Nikaido H. 2003. Molecular basis of bacterial outer membrane permeability revisited. Microbiol Mol Biol Rev 67:593–656. doi:10.1128/MMBR.67.4.593-656.2003. 14665678 PMC309051

[73] Ghosh M, Lin YM, Miller PA, Möllmann U, Boggess WC, Miller MJ. 2018. Siderophore conjugates of daptomycin are potent inhibitors of carbapenem resistant strains of *Acinetobacter baumannii*. ACS Infect Dis 4:1529–1535. doi:10.1021/acsinfecdis.8b00150. 30043609

[74] Meng J, Da F, Ma X, Wang N, Wang Y, Zhang H, Li M, Zhou Y, Xue X, Hou Z, Jia M, Luo X. 2015. Antisense growth inhibition of methicillin-resistant *Staphylococcus aureus* by locked nucleic acid conjugated with cell-penetrating peptide as a novel FtsZ inhibitor. Antimicrob Agents Chemother 59:914–922. doi:10.1128/AAC.03781-14. 25421468 PMC4335876

[75] Parker EN, Drown BS, Geddes EJ, Lee HY, Ismail N, Lau GW, Hergenrother PJ. 2020. Implementation of permeation rules leads to a FabI inhibitor with activity against Gram-negative pathogens. Nat Microbiol 5:67–75. doi:10.1038/s41564-019-0604-5. 31740764 PMC6953607

[76] Kanehisa M, Goto S. 2000. KEGG: kyoto encyclopedia of genes and genomes. Nucleic Acids Res 28:27–30. doi:10.1093/nar/28.1.27. 10592173 PMC102409

[77] Li J, Yao Y, Xu HH, Hao L, Deng Z, Rajakumar K, Ou HY. 2015. SecReT6: a web-based resource for type VI secretion systems found in bacteria. Environ Microbiol 17:2196–2202. doi:10.1111/1462-2920.12794. 25640659

[78] Barret M, Egan F, Fargier E, Morrissey JP, O'Gara F. 2011. Genomic analysis of the type VI secretion systems in *Pseudomonas* spp.: novel clusters and putative effectors uncovered. Microbiology (Reading) 157:1726–1739. doi:10.1099/mic.0.048645-0. 21474537

[79] Barret M, Egan F, O'Gara F. 2013. Distribution and diversity of bacterial secretion systems across metagenomic datasets. Environ Microbiol Rep 5:117–126. doi:10.1111/j.1758-2229.2012.00394.x. 23757140

[80] Suzek BE, Wang Y, Huang H, McGarvey PB, Wu CH, UniProt Consortium. 2015. UniRef clusters: a comprehensive and scalable alternative for improving sequence similarity searches. Bioinformatics 31:926–932. doi:10.1093/bioinformatics/btu739. 25398609 PMC4375400

[81] Camacho C, Madden T, Tao T, Agarwala R, Morgulis A. 2008. BLAST® command line applications user manual. National Center for Biotechnology Information, Bethesda, MD. https://www.ncbi.nlm.nih.gov/books/NBK279690/. Accessed February 2020.

[82] Pasolli E, Asnicar F, Manara S, Zolfo M, Karcher N, Armanini F, Beghini F, Manghi P, Tett A, Ghensi P, Collado MC, Rice BL, DuLong C, Morgan XC, Golden CD, Quince C, Huttenhower C, Segata N. 2019. Extensive unexplored human microbiome diversity revealed by over 150,000 genomes from metagenomes spanning age, geography, and lifestyle. Cell 176:649–662.e20. doi:10.1016/j.cell.2019.01.001. 30661755 PMC6349461

[83] Woodcroft BJ, Boyd JA, Tyson GW. 2016. OrfM: a fast open reading frame predictor for metagenomic data. Bioinformatics 32:2702–2703. doi:10.1093/bioinformatics/btw241. 27153669 PMC5013905

[84] Li W, Jaroszewski L, Godzik A. 2001. Clustering of highly homologous sequences to reduce the size of large protein databases. Bioinformatics 17:282–283. doi:10.1093/bioinformatics/17.3.282. 11294794

[85] Edgar RC. 2004. MUSCLE: multiple sequence alignment with high accuracy and high throughput. Nucleic Acids Res 32:1792–1797. doi:10.1093/nar/gkh340. 15034147 PMC390337

[86] Chiner-Oms A, González-Candelas F. 2016. EvalMSA: a program to evaluate multiple sequence alignments and detect outliers. Evol Bioinform Online 12:277–284. doi:10.4137/EBO.S40583. 27920488 PMC5127606

[87] Waterhouse AM, Procter JB, Martin DM, Clamp M, Barton GJ. 2009. JalView version 2—a multiple sequence alignment editor and analysis workbench. Bioinformatics 25:1189–1191. doi:10.1093/bioinformatics/btp033. 19151095 PMC2672624

[88] Henikoff S, Henikoff JG. 1992. Amino acid substitution matrices from protein blocks. PSroc Natl Acad Sci U S A 89:10915–10919. doi:10.1073/pnas.89.22.10915. PMC50453 1438297

[89] Pettersen EF, Goddard TD, Huang CC, Couch GS, Greenblatt DM, Meng EC, Ferrin TE. 2004. UCSF Chimera—a visualization system for exploratory research and analysis. J Comput Chem 25:1605–1612. doi:10.1002/jcc.20084. 15264254

[90] Crooks GE, Hon G, Chandonia JM, Brenner SE. 2004. WebLogo: a sequence logo generator. Genome Res 14:1188–1190. doi:10.1101/gr.849004. 15173120 PMC419797

[91] Waskom M. 2017. mwaskom/seaborn: v0.8.1 (September 2017). Zenodo. 10.5281/zenodo.883859.

[92] Brooks BR, Brooks CL, 3rd, Mackerell AD, Jr, Nilsson L, Petrella RJ, Roux B, Won Y, Archontis G, Bartels C, Boresch S, Caflisch A, Caves L, Cui Q, Dinner AR, Feig M, Fischer S, Gao J, Hodoscek M, Im W, Kuczera K, Lazaridis T, Ma J, Ovchinnikov V, Paci E, Pastor RW, Post CB, Pu JZ, Schaefer M, Tidor B, Venable RM, Woodcock HL, Wu X, Yang W, York DM, Karplus M. 2009. CHARMM: the biomolecular simulation program. J Comput Chem 30:1545–1614. doi:10.1002/jcc.21287. 19444816 PMC2810661

[93] Wang S, Sun S, Li Z, Zhang R, Xu J. 2017. Accurate de novo prediction of protein contact map by ultra-deep learning model. PLoS Comput Biol 13:e1005324. doi:10.1371/journal.pcbi.1005324. 28056090 PMC5249242

[94] Ovchinnikov S, Kamisetty H, Baker D. 2014. Robust and accurate prediction of residue-residue interactions across protein interfaces using evolutionary information. ELife 3:e02030. doi:10.7554/eLife.02030. 24842992 PMC4034769

[95] Li MZ, Elledge SJ. 2007. Harnessing homologous recombination in vitro to generate recombinant DNA via SLIC. Nat Methods 4:251–256. doi:10.1038/nmeth1010. 17293868

[96] Jeong JY, Yim HS, Ryu JY, Lee HS, Lee JH, Seen DS, Kang SG. 2012. One-step sequence- and ligation-independent cloning as a rapid and versatile cloning method for functional genomics studies. Appl Environ Microbiol 78:5440–5443. doi:10.1128/AEM.00844-12. 22610439 PMC3416421

[97] Brunet YR, Espinosa L, Harchouni S, Mignot T, Cascales E. 2013. Imaging type VI secretion-mediated bacterial killing. Cell Rep 3:36–41. doi:10.1016/j.celrep.2012.11.027. 23291094

[98] Zoued A, Durand E, Bebeacua C, Brunet YR, Douzi B, Cambillau C, Cascales E, Journet L. 2013. TssK is a trimeric cytoplasmic protein interacting with components of both phage-like and membrane anchoring complexes of the type VI secretion system. J Biol Chem 288:27031–27041. doi:10.1074/jbc.M113.499772. 23921384 PMC3779704

[99] Link AJ, Phillips D, Church GM. 1997. Methods for generating precise deletions and insertions in the genome of wild-type *Escherichia coli*: application to open reading frame characterization. J Bacteriol 179:6228–6237. doi:10.1128/jb.179.20.6228-6237.1997. 9335267 PMC179534

[100] Flaugnatti N, Le TT, Canaan S, Aschtgen MS, Nguyen VS, Blangy S, Kellenberger C, Roussel A, Cambillau C, Cascales E, Journet L. 2016. A phospholipase A1 antibacterial type VI secretion effector interacts directly with the C-terminal domain of the VgrG spike protein for delivery. Mol Microbiol 99:1099–1118. doi:10.1111/mmi.13292. 26714038

[101] Gueguen E, Cascales E. 2013. Promoter swapping unveils the role of the *Citrobacter rodentium* CTS1 type VI secretion system in interbacterial competition. Appl Environ Microbiol 79:32–38. doi:10.1128/AEM.02504-12. 23064344 PMC3536073

[102] Schneider CA, Rasband WS, Eliceiri KW. 2012. NIH Image to ImageJ: 25 years of image analysis. Nat Methods 9:671–675. doi:10.1038/nmeth.2089. 22930834 PMC5554542

[103] Ducret A, Quardokus EM, Brun YV. 2016. MicrobeJ, a tool for high throughput bacterial cell detection and quantitative analysis. Nat Microbiol 1:16077. doi:10.1038/nmicrobiol.2016.77. 27572972 PMC5010025

