# Supplementary figures and images for: Inhibiting Type VI Secretion System Activity with a Biomimetic Peptide Designed To Target the Baseplate Wedge Complex
Source: mBio. 2021 Aug 10;12(4):e01348-21. doi: 10.1128/mBio.01348-21 (PMC8406304; doi:10.1128/mBio.01348-21)

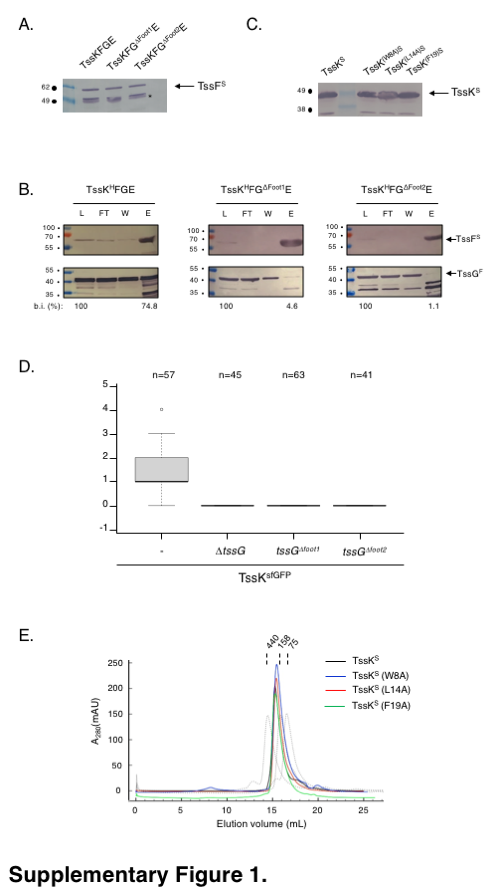

Supplement: FIG S1 [file mbio.01348-21-sf001.tif]

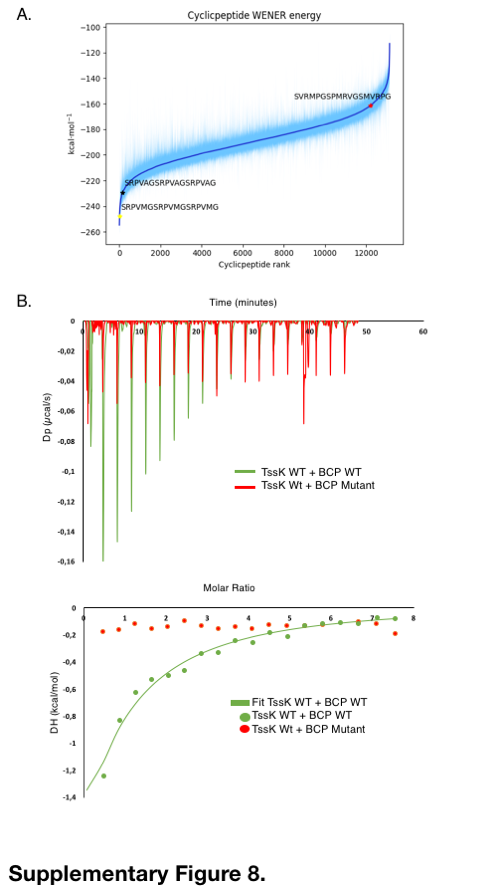

Supplement: FIG S8 [file mbio.01348-21-sf008.tif]

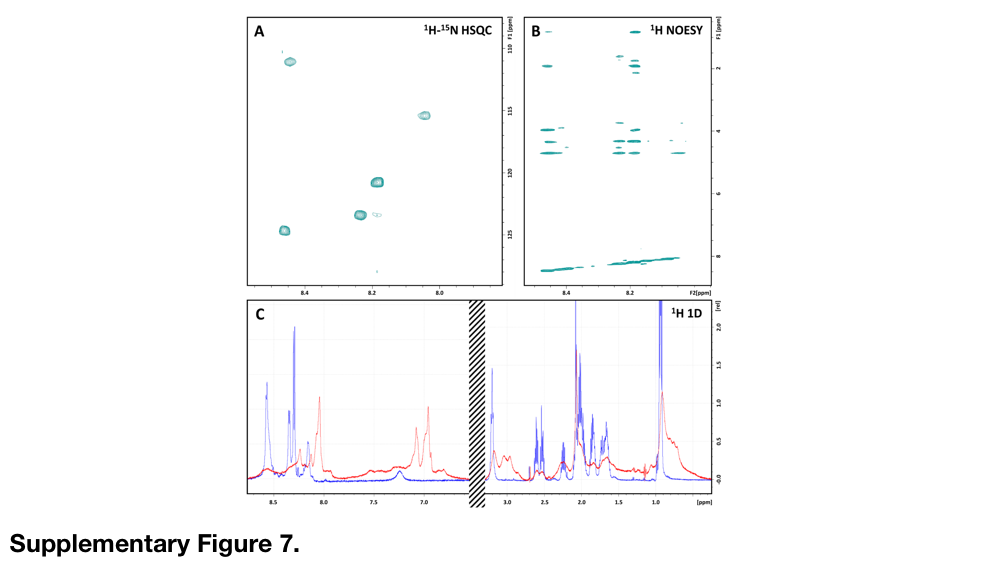

Supplement: FIG S7 [file mbio.01348-21-sf007.tif]

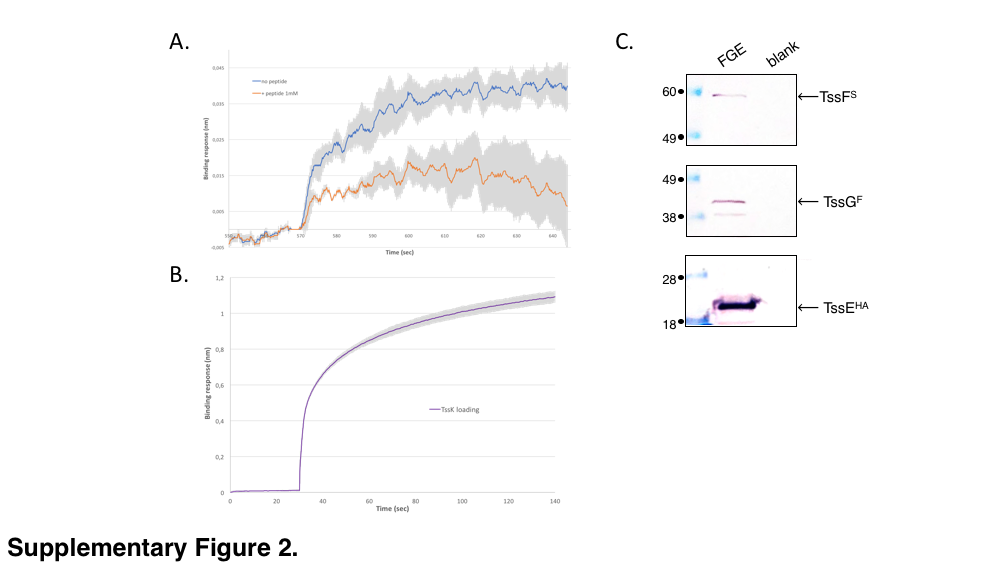

Supplement: FIG S2 [file mbio.01348-21-sf002.tif]

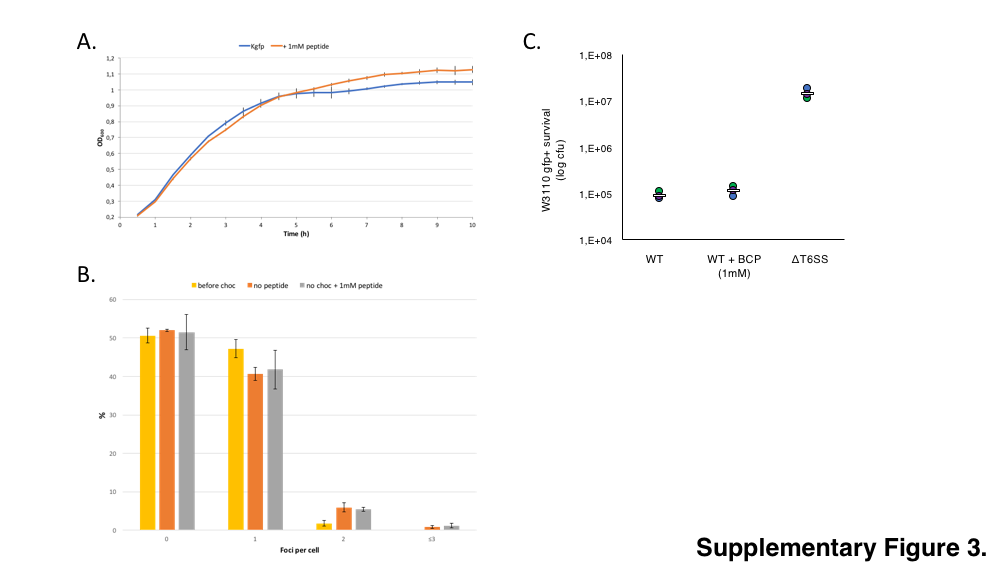

Supplement: FIG S3 [file mbio.01348-21-sf003.tif]

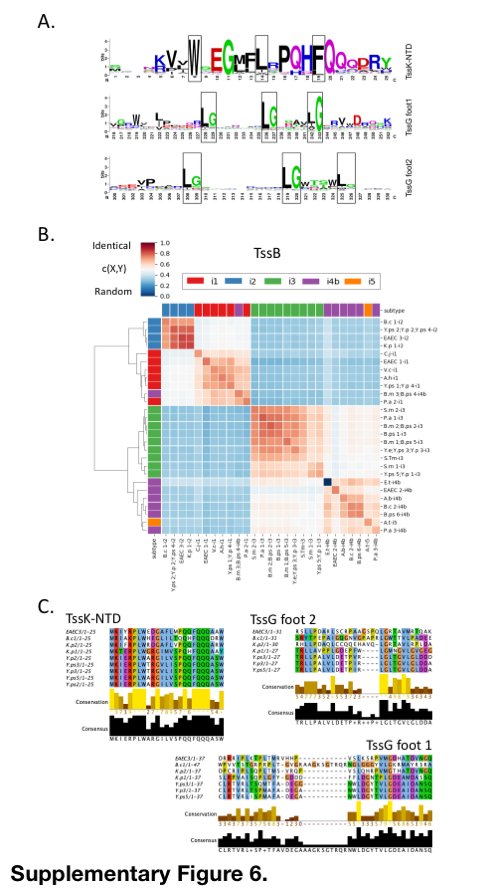

Supplement: FIG S6 [file mbio.01348-21-sf006.tif]

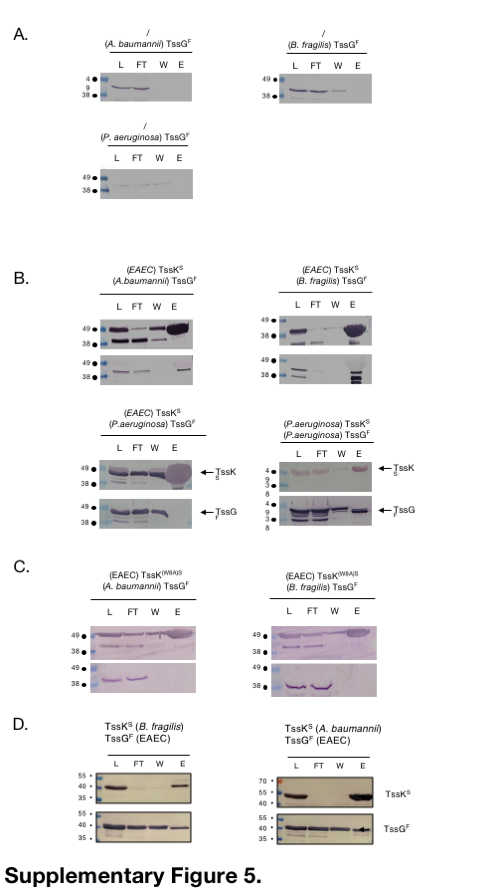

Supplement: FIG S5 [file mbio.01348-21-sf005.tif]

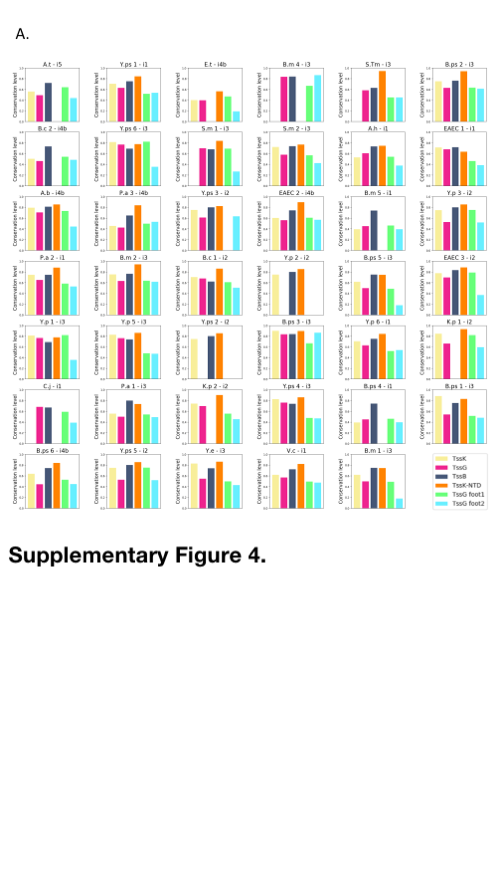

Supplement: FIG S4 [file mbio.01348-21-sf004.tif]

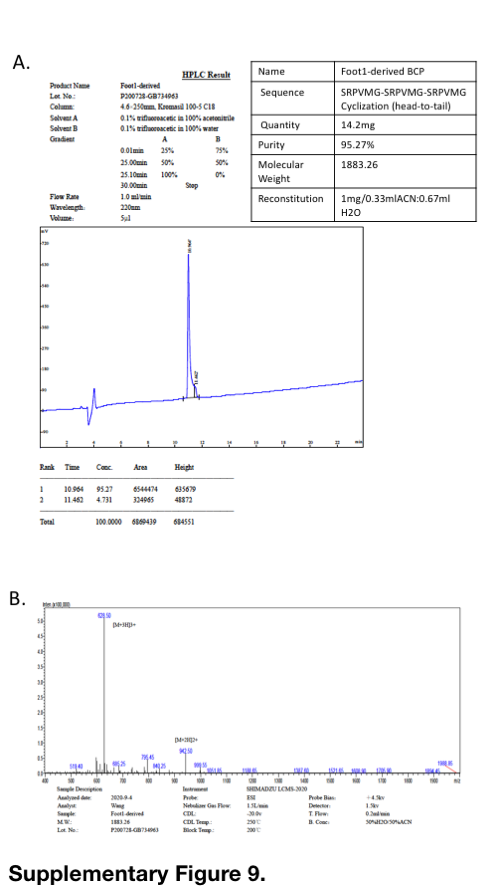

Supplement: FIG S9 [file mbio.01348-21-sf009.tif]
