# Supplementary material for: Inhibiting Type VI Secretion System Activity with a Biomimetic Peptide Designed To Target the Baseplate Wedge Complex
Source: mBio. 2021 Aug 10;12(4):e01348-21. doi: 10.1128/mBio.01348-21 (PMC8406304; doi:10.1128/mBio.01348-21)
Supplement: TABLE S1 [file mbio.01348-21-st001.docx]

**SUPPLEMENTAL TABLE S1A. Strains, plasmids and oligonucleotides used in this study.**

**Strains**

**Strains Description and genotype Source**

*E. coli* K12

DH5α F-, Δ(*argF*-l*ac*)U169, *phoA*, s*upE44*, Δ(lac*Z*)M15, *relA*, *endA*, *thi*, *hsdR*  Laboratory collection

BL21 (DE3) *fhuA2 [lon] ompT gal (λ DE3) [dcm] ∆hsdS λ DE3 = λ sBamHIo∆EcoRI-B New England Biolabs*

*int::(lacI::PlacUV5::T7 gene1) i21 ∆nin5*

W3110  *F-, lambda-IN(rrnD-rrnE)1 rph-1*  Laboratory collection

Enteroaggregative *E. coli*

17-2 Wild-type enteroaggregative *Escherichia coli* Arlette Darfeuille-Michaud

17-2Δ*tssG* 17.2 strain deleted of the *tss*G gene Brunet *et al*., 2015

17-2Δ*tssK* 17.2 strain deleted of the *tss*K gene Zoued *et al*., 2013

17-2 *tssK*-*gfp* gfp-mut2 inserted upstream the stop codon of *tssK* in 17-2 Brunet *et al*., 2015

17-2 *tssK*-*gfp*Δ*tssG* 17.2 *tssK*-gfp strain deleted of the *tssG* gene Cherrak *et al*., 2018

17-2 *tssG*^Δfoot1^ 17.2 strain deleted of the foot1 (216-252) sequence located in the *tssG* gene This study

17-2 *tssG*^Δfoot2^ 17.2 strain deleted of the foot2 sequence (300-330) located in the *tssG* gene This study

17-2 *tssK*-*gfp* *tssG*^Δfoot1^ 17.2 *tssK*-gfp strain deleted of the foot1 (216-252) sequence located in the *tssG* gene This study

17-2 *tssK*-*gfp* *tssG*^Δfoot2^ 17.2 *tssK*-gfp deleted of the foot2 (300-330) sequence located in the *tssG* gene This study

**Plasmids**

**Vectors Description Source**

Vectors for chromosomal insertions

pKO3 Suicide vector for allelic replacement Link *et al*, 1997

pKO3-*tssG*-C-term pKO3 vector encoding the TssG sequence targeted by mutagenesis This study

pKO3-*tssG*^Δfoot1^ pKO3-*tssG*-C-term vector deleted of the foot 1 sequence This study

pKO3-*tssG*^Δfoot2^ pKO3-*tssG*-C-term vector deleted of the foot 2 sequence This study

Expression vectors

pTRC-99A cloning vector, pBR322 origin, lacI, AmpR Laboratory collection

pTRC-99A-TssK^H^ *sci1* *tssK* sequence cloned into pTRC-99A, C-terminal HIS epitope Laboratory collection

pTRC-99A-TssK^(W8A)H^ pTRC-99A-TssK^H^ harboring a Trp→Ala mutation in position 8 This study

pTRC-99A-TssK^(L14A)H^ pTRC-99A-TssK^H^ harboring a Leu→Ala mutation in position 14 This study

pTRC-99A-TssK^(F19A)H^ pTRC-99A-TssK^H^ harboring a Phe→Ala mutation in position 19 This study

Vectors for protein overproduction

pCDF-Duet1 Expression vector, lacI, PT7, SM^R^ Addgene

pRSF-Duet1 Expression vector, lacI, PT7, Kan^R^ Addgene

pCDF-TssK^H^-^S^F-G^Flag^-^HA^E *tssK-*6xHIS, StrepII-*tssF*, *tssG*-FLAG, HA-*tssE* cloned into pCDF-Duet1 Cherrak *et al*., 2018

pCDF-TssK^H^ *tssK-*6xHIS cloned into pCDF-Duet1 Cherrak *et al*., 2018

pCDF-TssG^Flag^ *tssG*-FLAG cloned into pCDF-Duet1 Cherrak *et al*., 2018

pCDF-TssK^H^-^S^F-G^Δfoot1Flag^-^HA^E *tssK-*6xHIS, StrepII-*tssF*, *tssG* ^Δfoot1^-FLAG, HA-*tssE* cloned into pCDF-Duet1 This study

pCDF-TssK^H^-^S^F-G^Δfoot2Flag^-^HA^E *tssK-*6xHIS, StrepII-*tssF*, *tssG* ^Δfoot2^-FLAG, HA-*tssE* cloned into pCDF-Duet1 This study

pRSF-TssK^S^ *tssK-*StrepII cloned into pRSF-Duet1 This study

pRSF-TssK^(W8A)S^ pRSF-TssK^S^ harboring a Trp→Ala mutation in position 8 This study

pRSF-TssK^(L14A)S^ pRSF-TssK^S^ harboring a Leu→Ala mutation in position 14 This study

pRSF-TssK^(F19A)S^ pRSF-TssK^S^ harboring a Phe→Ala mutation in position 19 This study

pRSF-TssK^(F19L)S^ pRSF-TssK^S^ harboring a Phe→Leu mutation in position 19 This study

pRSF-TssK^S(AB)^ *Acinetobacter baumannii* *tssK-*StrepII cloned into pRSF-Duet1 This study

pCDF-TssG^Flag(AB)^ *Acinetobacter baumannii* *tssG-*FLAG cloned into pCDF-Duet1 This study

pRSF-TssK^S(PA)^ *Pseudomonas aeruginosa* (HSI-2) *tssK-*StrepII cloned into pRSF-Duet1 This study

pCDF-TssG^Flag(PA)^ *Pseudomonas aeruginosa* (HSI-2) *tssG-*FLAG cloned into pCDF-Duet1 This study

pRSF-TssK^S(^*^Bf^*^)^ *Bacteroides fragilis* *tssK-*StrepII cloned into pRSF-Duet1 This study

pCDF-TssG^Flag(^*^Bf^*^)^ *Bacteroides fragilis tssG-*FLAG cloned into pCDF-Duet1 This study

**Oligonucleotides** ^a, b, c^

**Name Destination Sequence (5’**→**3’)**

For allelic replacement (pKO3)

pKO3-*tssG*-C-term *sci1 tssG* residues (168-end) flanked by 600 nucleotides uptstream and dowtreasm, cloned into pKO3

FWD: ATT**GCGGCCGC**CCTGGCCCTGTACGCCGATATGAATC

REV: ATT**GCGGCCGC**GCTCTTCCTCACCGACCTGGTTTAAC

pKO3-*tssG*^Δfoot1^ Deletion of the foot1 sequence using the plasmid pKO3-*tssG*-C-term as matrix

FWD1: GGAACACAGGCAGACGGGCAGCGAGGTACA

REV1: GCTTCTCAAATGCCTCAGGC

FWD2: GCCTGAGGCATTTGAGAAGC

REV2: TGCCCGTCTGCTGTGTTCCGGGAGCCAGCA

pKO3-*tssG*^Δfoot2^ Deletion of the foot2 sequence using the plasmid pKO3-*tssG*-C-term as matrix

FWD1: TCTGGGAAGCCGGGTTCAGGAACATTATCAACGT

REV1: GCTTCTCAAATGCCTCAGGC

FWD2: GCCTGAGGCATTTGAGAAGC

REV2: CCTGAACCCGGCTTCCCAGATAAACATGCAACAACG

For complementation experiments (pTRC-99A)

pTRC-99A-TssK^(W8A)H^ Mutagenesis of the pTRC-99A-TssK^H^ to introduce a Trp→Ala substitution in position 8

FWD: CAGACCATGAAGATTTATCGCCCATTAGCGGAAGACGGGGCTTTTCTGATGCC

REV: GGCATCAGAAAAGCCCCGTCTTCCGCTAATGGGCGATAAATCTTCATGGTCTG

pTRC-99A-TssK^(L14A)H^ Mutagenesis of the pTRC-99A-TssK^H^ to introduce a Leu→Ala substitution in position 14

FWD: GGGAAGACGGGGCTTTTGCGCTGATGCCCCAGCAGTTTC

REV: GAAACTGCTGGGGCATCAGCGCAAAAGCCCCGTCTTCCC

pTRC-99A-TssK^(F19A)H^ Mutagenesis of the pTRC-99A-TssK^H^ to introduce a Phe→Ala substitution in position 19

FWD: GCTTTTCTGATGCCCCAGCAGGCGCAGCAGCAGGCTGCCTGGG

REV: CCCAGGCAGCCTGCTGCTGCGCCTGCTGGGGCATCAGAAAAGC

For biochemistry experiments (pCDF/pRSF)

pCDF-TssK^H^-^S^F-G^Δfoot1Flag^-^HA^E Insertion of the *tssK-*6xHIS, StrepII-*tssF*, *tssG* ^Δfoot1^-FLAG, HA-*tssE* into pCDF-Duet using plasmid pCDF-TssK^H^-^S^F-G^Flag^-

^HA^E as matrix

FWD1: GGAACACAGGCAGACGGGCAGCGAGGTACA

REV1: GGGATACGACGATACCGAAGACA

FWD2: TGTCTTCGGTATCGTCGTATCCC

REV2: TGCCCGTCTGCTGTGTTCCGGGAGCCAGCA

pCDF-TssK^H^-^S^F-G^Δfoo21Flag^-^HA^E Insertion of the *tssK-*6xHIS, StrepII-*tssF*, *tssG* ^Δfoot2^-FLAG, HA-*tssE* into pCDF-Duet using plasmid pCDF-TssK^H^-^S^F-G^Flag^-^HA^E as matrix

FWD1: TCTGGGAAGCCGGGTTCAGGAACATTATCAACGT

REV1: GGGATACGACGATACCGAAGACA

FWD2: TGTCTTCGGTATCGTCGTATCCC

REV2: CCTGAACCCGGCTTCCCAGATAAACATGCAACAACG

pRSF-TssK^S^ Insertion of the *EAEC sci1* *tssK* sequence into pRSF-Duet, C-terminal Strep-II epitope

FWD1: TAATTTTGTTTAACTTTAATAAGGAGATATACCATGAAGATTTATCGCCCATTATGGGAAGACG

REV1: TTTCTGTTCGACTTAAGCATTATGCGGCCGCTCA*TTTTTCGAACTGCGGGTGGCTCCA*TGTCCGCAGCACCGCAAAAAGTTC

pRSF-TssK^(W8A)S^  Mutagenesis of the pRSF-K^S^ to introduce a Trp→Ala substitution in position 8

FWD: CAGACCATGAAGATTTATCGCCCATTAGCGGAAGACGGGGCTTTTCTGATGCC

REV: GGCATCAGAAAAGCCCCGTCTTCCGCTAATGGGCGATAAATCTTCATGGTCTG

pRSF-TssK^(L14A)S^  Mutagenesis of the pRSF-K^S^ to introduce a Leu→Ala substitution in position 14

FWD: GGGAAGACGGGGCTTTTGCGCTGATGCCCCAGCAGTTTC

REV: GAAACTGCTGGGGCATCAGCGCAAAAGCCCCGTCTTCCC

pRSF-TssK^(F19A)S^  Mutagenesis of the pRSF-K^S^ to introduce a Phe→Ala substitution in position 19

FWD: GCTTTTCTGATGCCCCAGCAGGCGCAGCAGCAGGCTGCCTGGG

REV: CCCAGGCAGCCTGCTGCTGCGCCTGCTGGGGCATCAGAAAAGC

pRSF-TssK^(F19L)S^  Mutagenesis of the pRSF-K^S^ to introduce a Phe→Leu substitution in position 19

FWD: GCTTTTCTGATGCCCCAGCAGCTGCAGCAGCAGGCTGCCTGGG

REV: CCCAGGCAGCCTGCTGCTGCAGCTGCTGGGGCATCAGAAAAGC

pRSF-TssK^S(AB)^ Insertion of the *Acinetobacter baumannii* *tssK* sequence into pRSF-Duet, C-terminal Strep-II epitope

FWD1: TTTAACTTTAATAAGGAGATATACCATGAACGTGCACAAGATCGTCTGGC

REV1: GATGGTGATGGCTGCTGCCTCA*TTTTTCGAACTGCGGGTGGCTCCA*GTTCCTGATGGCCCAGAATTTCAGTTC

FWD2: GGCAGCAGCCATCACCATC

REV2: CATGGTATATCTCCTTATTAAAGTTAAA

pCDF-TssG^Flag(AB)^ Insertion of the *Acinetobacter baumannii* *tssG* sequence into pCDF-Duet, C-terminal FLAG epitope

FWD1: TTTAACTTTAATAAGGAGATATACCATGCACTCAGAACGTTGGTGGCAAGAC

REV1: GATGGTGATGGCTGCTGCCTCA*CTTGTCATCGTCATCTTTATAATC*TATTTGCTCTCCTATAAGGCTATAACATGTTTC

FWD2: GGCAGCAGCCATCACCATC

REV2: CATGGTATATCTCCTTATTAAAGTTAAA

pCDF-TssK^S(PA)^ Insertion of the *Pseudomonas aeruginosa* (HSI-2) *tssK* sequence into pCDF-Duet, C-terminal Strep-II epitope

FWD1: TTTAACTTTAATAAGGAGATATACCATGAACGTGCACAAGATCGTCTGGC

REV1: GATGGTGATGGCTGCTGCCTCA*TTTTTCGAACTGCGGGTGGCTCCA*GTTCCTGATGGCCCAGAATTTCAGTTC

FWD2: GGCAGCAGCCATCACCATC

REV2: CATGGTATATCTCCTTATTAAAGTTAAA

pRSF-TssG^Flag(PA)^ Insertion of the *Pseudomonas aeruginosa* (HSI-2) *tssG* sequence into pRSF-Duet, C-terminal Flag-II epitope

FWD1: TTTAACTTTAATAAGGAGATATACCATGGAAACCGCGCATGGGTTTG

REV1: GATGGTGATGGCTGCTGCCTTA*CTTGTCATCGTCATCTTTATAATC*ATGAATCTTGCTGCCCAGCGTCAC

FWD2: GGCAGCAGCCATCACCATC

REV2: CATGGTATATCTCCTTATTAAAGTTAAA

^b^ sequence annealing to the target vector underlined

^c^ FLAG or StrepII tag coding sequence *italicized*

^d^ restriction site in **bold**

**REFERENCES**

Brunet, Y. R., Zoued, A., Boyer, F., Douzi, B., & Cascales, E. (2015). The type VI secretion TssEFGK-VgrG phage-like baseplate is recruited to the TssJLM membrane complex via multiple contacts and serves as assembly platform for tail tube/sheath polymerization. *PLoS genetics*, *11*(10), e1005545.

Cherrak, Y., Rapisarda, C., Pellarin, R., Bouvier, G., Bardiaux, B., Allain, F., ... & Durand, E. (2018). Biogenesis and structure of a type VI secretion baseplate. *Nature microbiology*, *3*(12), 1404-1416.

Link, A. J., Phillips, D., & Church, G. M. (1997). Methods for generating precise deletions and insertions in the genome of wild-type Escherichia coli: application to open reading frame characterization. *Journal of bacteriology*, *179*(20), 6228-6237.

Zoued, A., Durand, E., Bebeacua, C., Brunet, Y. R., Douzi, B., Cambillau, C., ... & Journet, L. (2013). TssK is a trimeric cytoplasmic protein interacting with components of both phage-like and membrane anchoring complexes of the type VI secretion system. *Journal of Biological Chemistry*, *288*(38), 27031-27041.

**SUPPLEMENTAL TABLE S1B. Bacterial strains naming for the *in silico* approach.**

| **Bacteria** | **Acronym** | **T6SS subtype** |
| --- | --- | --- |
| *Enteroaggregative Escherichia coli* | EAEC1 | i1 |
|  | EAEC2 | i4b |
|  | EAEC3 | i2 |
| *Acinetobacter baumannii* | A. b | i4b |
| *Vibrio cholerae* | V. c | i1 |
| *Pseudomonas aeruginosa* | P. a 1 | i3 |
|  | P. a 2 | i1 |
|  | P. a 3 | i4b |
| *Serratia marcescens* | S. m 1 | i3 |
|  | S. m 2 | i3 |
| *Salmonella cnterica Serovar Typhimurium* | S. tm | i3 |
| *Klebsiella pneumonia* | K. p 1 | i2 |
|  | K. p 2 | i2 |
| *Aeromonas hydrophila* | A. h | i1 |
| *Campylobacter jejuni* | C. j | i1 |
| *Yersinia enterocolitica* | Y. e | i3 |
| *Yersinia pseudotuberculosis* | Y. ps 1 | i1 |
|  | Y. ps 2 | i2 |
|  | Y. ps 3 | i2 |
|  | Y. ps 4 | i3 |
|  | Y. ps 5 | i2 |
|  | Y. ps 6 | i3 |
| *Yersinia pestis* | Y. p 1 | i3 |
|  | Y. p 2 | i2 |
|  | Y. p 3 | i2 |
|  | Y. p 4 | i3 |
|  | Y. p 5 | i3 |
|  | Y. p 6 | i1 |
| *Edwardsiella tarda* | E. t | i4b |
| *Agrobacterium tumefaciens* | A. t | i5 |
| *Burkholderia cenocepacia* | B. c 1 | i2 |
|  | B. c 2 | i4b |
| *Burkholderia mallei* | B. m 1 | i3 |
|  | B. m 2 | i3 |
|  | B. m 3 | i4b |
|  | B. m 4 | i3 |
|  | B. m 5 | i1 |
| *Burkholderia pseudomallei* | B. ps 1 | i3 |
|  | B. ps 2 | i3 |
|  | B. ps 3 | i3 |
|  | B. ps 4 | i1 |
|  | B. ps 5 | i3 |
|  | B. ps 6 | i4b |

**SUPPLEMENTAL TABLE S1C. Protein sequences for the *in silico* approach.**

| **Sequence** | **GB accession no.** | **Cluster id** | **T6SS subtype** | **Author** |
| --- | --- | --- | --- | --- |
| EAEC1 TssK | CAU96104 | 241895..263191 | i1 | Genoscope - Centre National de Sequencage : BP 191 91006 EVRY cedex - FRANCE. 2008 |
| EAEC1 TssG | CAU96107 | 241895..263191 | i1 | Genoscope - Centre National de Sequencage : BP 191 91006 EVRY cedex - FRANCE. 2008 |
| EAEC1 TssB | CAU96112 | 241895..263191 | i1 | Genoscope - Centre National de Sequencage : BP 191 91006 EVRY cedex - FRANCE. 2008 |
| EAEC2 TssK | CAU99307 | 3369231..3387598 | i4b | Genoscope - Centre National de Sequencage : BP 191 91006 EVRY cedex - FRANCE. 2008 |
| EAEC2 TssG | CAU99318 | 3369231..3387598 | i4b | Genoscope - Centre National de Sequencage : BP 191 91006 EVRY cedex - FRANCE. 2008 |
| EAEC2 TssB | CAU99327 | 3369231..3387598 | i4b | Genoscope - Centre National de Sequencage : BP 191 91006 EVRY cedex - FRANCE. 2008 |
| EAEC3 TssK | CAU99391 | 3394967..3428639 | i2 | Genoscope - Centre National de Sequencage : BP 191 91006 EVRY cedex - FRANCE. 2008 |
| EAEC3 TssG | CAU99363 | 3394967..3428639 | i2 | Genoscope - Centre National de Sequencage : BP 191 91006 EVRY cedex - FRANCE. 2008 |
| EAEC3 TssB | CAU99394 | 3394967..3428639 | i2 | Genoscope - Centre National de Sequencage : BP 191 91006 EVRY cedex - FRANCE. 2008 |
| A. b TssK | AHB91894 | 2353802..2383994 | i4b | X. Wang et al. Unpublished. 2013 |
| A. b TssG | AHB91902 | 2353802..2383994 | i4b | X. Wang et al. Unpublished. 2013 |
| A. b TssB | AHB91907 | 2353802..2383994 | i4b | X. Wang et al. Unpublished. 2013 |
| V. c TssK | ACP07093 | 115133..141582 | i1 | L. Feng et al. PLoS ONE. 2008. 3 (12), E4053 |
| V. c TssG | ACP07090 | 115133..141582 | i1 | L. Feng et al. PLoS ONE. 2008. 3 (12), E4053 |
| V. c TssB | ACP07086 | 115133..141582 | i1 | L. Feng et al. PLoS ONE. 2008. 3 (12), E4053 |
| P. a 1 TssK | AGY66194 | 90844..117524 | i3 | Y. Yin et al. Genome Announc. 2013. e01031-13 |
| P. a 1 TssG | AGY66614 | 90844..117524 | i3 | Y. Yin et al. Genome Announc. 2013. e01031-13 |
| P. a 1 TssB | AGY63037 | 90844..117524 | i3 | Y. Yin et al. Genome Announc. 2013. e01031-13 |
| P. a 2 TssK | AGY66073 | 1803621..1822596 | i1 | Y. Yin et al. Genome Announc. 2013. e01031-13 |
| P. a 2 TssG | AGY64631 | 1803621..1822596 | i1 | Y. Yin et al. Genome Announc. 2013. e01031-13 |
| P. a 2 TssB | AGY68344 | 1803621..1822596 | i1 | Y. Yin et al. Genome Announc. 2013. e01031-13 |
| P. a 3 TssK | AGY63073 | 2607127..2626205 | i4b | Y. Yin et al. Genome Announc. 2013. e01031-13 |
| P. a 3 TssG | AGY68374 | 2607127..2626205 | i4b | Y. Yin et al. Genome Announc. 2013. e01031-13 |
| P. a 3 TssB | AGY63622 | 2607127..2626205 | i4b | Y. Yin et al. Genome Announc. 2013. e01031-13 |
| S. m 1 TssK | AIA48015 | 2826850..2855641 | i3 | P. Li et al. PLoS ONE. 2015. 10 (4), E0123061 |
| S. m 1 TssG | AIA48007 | 2826850..2855641 | i3 | P. Li et al. PLoS ONE. 2015. 10 (4), E0123061 |
| S. m 1 TssB | AIA48002 | 2826850..2855641 | i3 | P. Li et al. PLoS ONE. 2015. 10 (4), E0123061 |
| S. m 2 TssK | AIA48190 | 3025547..3063156 | i3 | P. Li et al. PLoS ONE. 2015. 10 (4), E0123061 |
| S. m 2 TssG | AIA48172 | 3025547..3063156 | i3 | P. Li et al. PLoS ONE. 2015. 10 (4), E0123061 |
| S. m 2 TssB | AIA48185 | 3025547..3063156 | i3 | P. Li et al. PLoS ONE. 2015. 10 (4), E0123061 |
| S. Tm TssK | AGQ68936 | 3228066..3262201 | i3 | M. Hoffmann et al. Genome Announc. 2013. 1 (6), e01068-13 |
| S. Tm TssG | AGQ68948 | 3228066..3262201 | i3 | M. Hoffmann et al. Genome Announc. 2013. 1 (6), e01068-13 |
| S. Tm TssB | AGQ68943 | 3228066..3262201 | i3 | M. Hoffmann et al. Genome Announc. 2013. 1 (6), e01068-13 |
| K. p 1 TssK | BAH63077 | 2268445..2329098 | i2 | K. M. Wu et al. Unpublished. 2004. National Health Research Institutes, Division of Molecular and Genomic Medicine, Taiwan. |
| K. p 1 TssG | BAH63099 | 2268445..2329098 | i2 | K. M. Wu et al. Unpublished. 2004. National Health Research Institutes, Division of Molecular and Genomic Medicine, Taiwan. |
| K. p 1 TssB | BAH63050 | 2268445..2329098 | i2 | K. M. Wu et al. Unpublished. 2004. National Health Research Institutes, Division of Molecular and Genomic Medicine, Taiwan. |
| K. p 2 TssK | BAH63996 | 3185286..3215355 | i2 | K. M. Wu et al. Unpublished. 2004. National Health Research Institutes, Division of Molecular and Genomic Medicine, Taiwan. |
| K. p 2 TssG | BAH63978 | 3185286..3215355 | i2 | K. M. Wu et al. Unpublished. 2004. National Health Research Institutes, Division of Molecular and Genomic Medicine, Taiwan. |
| A. h TssK | AJE36729 | 2871129..2903427 | i1 | M. Pang et al. Unpublished. 2013.College of Veterinary Medicine, Nanjing Agricultural University |
| A. h TssG | AJE36732 | 2871129..2903427 | i1 | M. Pang et al. Unpublished. 2013.College of Veterinary Medicine, Nanjing Agricultural University |
| A. h TssB | AJE36736 | 2871129..2903427 | i1 | M. Pang et al. Unpublished. 2013.College of Veterinary Medicine, Nanjing Agricultural University |
| C. j TssK | AJK71139 | 964886..992379 | i1 | C. G. Clar et al. Unpublished. 2014. Enteric Diseases Program, National Microbiology Laboratory, Public Health Agency of Canada |
| C. j TssG | AJK71146 | 964886..992379 | i1 | C. G. Clar et al. Unpublished. 2014. Enteric Diseases Program, National Microbiology Laboratory, Public Health Agency of Canada |
| C. j TssB | AJK71142 | 964886..992379 | i1 | C. G. Clar et al. Unpublished. 2014. Enteric Diseases Program, National Microbiology Laboratory, Public Health Agency of Canada |
| Y. e TssK | AJJ27294 | 3532481..3566567 | i3 | S.L. Johnson et al. Genome Announc. 2015. 3 (2) |
| Y. e TssG | AJJ27880 | 3532481..3566567 | i3 | S.L. Johnson et al. Genome Announc. 2015. 3 (2) |
| Y. e TssB | AJJ27181 | 3532481..3566567 | i3 | S.L. Johnson et al. Genome Announc. 2015. 3 (2) |
| Y. ps 1 TssK | ABS46074 | 361733..385634 | i1 | M. Eppinger et al. PLoS Genet. 2007. 3 (8), E142 |
| Y. ps 1 TssG | ABS49816 | 361733..385634 | i1 | M. Eppinger et al. PLoS Genet. 2007. 3 (8), E142 |
| Y. ps 1 TssB | ABS46925 | 361733..385634 | i1 | M. Eppinger et al. PLoS Genet. 2007. 3 (8), E142 |
| Y. ps 2 TssK | ABS49416 | 884659..901538 | i2 | M. Eppinger et al. PLoS Genet. 2007. 3 (8), E142 |
| Y. ps 2 TssB | ABS46329 | 884659..901538 | i2 | M. Eppinger et al. PLoS Genet. 2007. 3 (8), E142 |
| Y. ps 3 TssK | ABS48268 | 932854..975730 | i2 | M. Eppinger et al. PLoS Genet. 2007. 3 (8), E142 |
| Y. ps 3 TssG | ABS49826 | 932854..975730 | i2 | M. Eppinger et al. PLoS Genet. 2007. 3 (8), E142 |
| Y. ps 3 TssB | ABS46901 | 932854..975730 | i2 | M. Eppinger et al. PLoS Genet. 2007. 3 (8), E142 |
| Y. ps 4 TssK | ABS48713 | 1587097..1619289 | i3 | M. Eppinger et al. PLoS Genet. 2007. 3 (8), E142 |
| Y. ps 4 TssG | ABS47559 | 1587097..1619289 | i3 | M. Eppinger et al. PLoS Genet. 2007. 3 (8), E142 |
| Y. ps 4 TssB | ABS46608 | 1587097..1619289 | i3 | M. Eppinger et al. PLoS Genet. 2007. 3 (8), E142 |
| Y. ps 5 TssK | ABS48245 | 2807319..2847574 | i2 | M. Eppinger et al. PLoS Genet. 2007. 3 (8), E142 |
| Y. ps 5 TssG | ABS49018 | 2807319..2847574 | i2 | M. Eppinger et al. PLoS Genet. 2007. 3 (8), E142 |
| Y. ps 5 TssB | ABS49130 | 2807319..2847574 | i2 | M. Eppinger et al. PLoS Genet. 2007. 3 (8), E142 |
| Y. ps 6 TssK | ABS49453 | 3850258..3875321 | i3 | M. Eppinger et al. PLoS Genet. 2007. 3 (8), E142 |
| Y. ps 6 TssG | ABS47917 | 3850258..3875321 | i3 | M. Eppinger et al. PLoS Genet. 2007. 3 (8), E142 |
| Y. ps 6 TssB | ABS47113 | 3850258..3875321 | i3 | M. Eppinger et al. PLoS Genet. 2007. 3 (8), E142 |
| Y. p 1 TssK | WP_002210470 | 531628..556690 | i3 | M. Kudryashev et al. Cell. 2015. 160 (5), 952-962 |
| Y. p 1 TssG | WP_002210476 | 531628..556690 | i3 | M. Kudryashev et al. Cell. 2015. 160 (5), 952-962 |
| Y. p 1 TssB | WP_002210481 | 531628..556690 | i3 | M. Kudryashev et al. Cell. 2015. 160 (5), 952-962 |
| Y. p 2 TssK | WP_002211664 | 1072398..1089082 | i2 | M. Kudryashev et al. Cell. 2015. 160 (5), 952-962 |
| Y. p 2 TssB | WP_002211662 | 1072398..1089082 | i2 | M. Kudryashev et al. Cell. 2015. 160 (5), 952-962 |
| Y. p 3 TssK | WP_002213011 | 1653684..1689928 | i2 | M. Kudryashev et al. Cell. 2015. 160 (5), 952-962 |
| Y. p 3 TssG | WP_002211941 | 1653684..1689928 | i2 | M. Kudryashev et al. Cell. 2015. 160 (5), 952-962 |
| Y. p 3 TssB | WP_002213014 | 1653684..1689928 | i2 | M. Kudryashev et al. Cell. 2015. 160 (5), 952-962 |
| Y. p 5 TssK | WP_002211571 | 3277633..3302045 | i3 | M. Kudryashev et al. Cell. 2015. 160 (5), 952-962 |
| Y. p 5 TssG | WP_002211583 | 3277633..3302045 | i3 | M. Kudryashev et al. Cell. 2015. 160 (5), 952-962 |
| Y. p 5 TssB | WP_002211575 | 3277633..3302045 | i3 | M. Kudryashev et al. Cell. 2015. 160 (5), 952-962 |
| Y. p 6 TssK | WP_002212105 | 4001721..4035636 | i1 | M. Kudryashev et al. Cell. 2015. 160 (5), 952-962 |
| Y. p 6 TssG | WP_002212102 | 4001721..4035636 | i1 | M. Kudryashev et al. Cell. 2015. 160 (5), 952-962 |
| Y. p 6 TssB | WP_002212098 | 4001721..4035636 | i1 | M. Kudryashev et al. Cell. 2015. 160 (5), 952-962 |
| E. t TssK | ACY85276 | 2554393..2580320 | i4b | Q. Wang et al. PLoS ONE. 2009. 4 (10), E7646 |
| E. t TssG | ACY85270 | 2554393..2580320 | i4b | Q. Wang et al. PLoS ONE. 2009. 4 (10), E7646 |
| E. t TssB | ACY85264 | 2554393..2580320 | i4b | Q. Wang et al. PLoS ONE. 2009. 4 (10), E7646 |
| A. t TssK | AKC10274 | 1998442..2033793 | i5 | Y. Y. Huang et al. Genome Announc. 2015. 3 (3), e00570-15 |
| A. t TssG | AKC10276 | 1998442..2033793 | i5 | Y. Y. Huang et al. Genome Announc. 2015. 3 (3), e00570-15 |
| A. t TssB | AKC10282 | 1998442..2033793 | i5 | Y. Y. Huang et al. Genome Announc. 2015. 3 (3), e00570-15 |
| B. c 1 TssK | CDN61992 | 172983..205070 | i2 | A.Carlier et al. Unpublished. 2014. Department of Microbiology, University of Zurich |
| B. c 1 TssG | CDN62003 | 172983..205070 | i2 | A.Carlier et al. Unpublished. 2014. Department of Microbiology, University of Zurich |
| B. c 1 TssB | CDN61993 | 172983..205070 | i2 | A.Carlier et al. Unpublished. 2014. Department of Microbiology, University of Zurich |
| B. c 2 TssK | CDN58852 | 347141..370099 | i4b | A.Carlier et al. Unpublished. 2014. Department of Microbiology, University of Zurich |
| B. c 2 TssG | CDN58860 | 347141..370099 | i4b | A.Carlier et al. Unpublished. 2014. Department of Microbiology, University of Zurich |
| B. c 2 TssB | CDN58855 | 347141..370099 | i4b | A.Carlier et al. Unpublished. 2014. Department of Microbiology, University of Zurich |
| B. m 1 TssK | AIO54269 | 161777..187715 | i3 | H. E. Daligault et al. Genome Announc. 2014. 2 (6) |
| B. m 1 TssG | AIO53426 | 161777..187715 | i3 | H. E. Daligault et al. Genome Announc. 2014. 2 (6) |
| B. m 1 TssB | AIO54802 | 161777..187715 | i3 | H. E. Daligault et al. Genome Announc. 2014. 2 (6) |
| B. m 2 TssK | AIO54049 | 793604..820431 | i3 | H. E. Daligault et al. Genome Announc. 2014. 2 (6) |
| B. m 2 TssG | AIO53893 | 793604..820431 | i3 | H. E. Daligault et al. Genome Announc. 2014. 2 (6) |
| B. m 2 TssB | AIO54309 | 793604..820431 | i3 | H. E. Daligault et al. Genome Announc. 2014. 2 (6) |
| B. m 4 TssG | AIO54573 | 972744..1000481 | i3 | H. E. Daligault et al. Genome Announc. 2014. 2 (6) |
| B. m 4 TssB | AIO50864 | 972744..1000481 | i3 | H. E. Daligault et al. Genome Announc. 2014. 2 (6) |
| B. m 5 TssK | AIO54625 | 1794749..1822794 | i1 | H. E. Daligault et al. Genome Announc. 2014. 2 (6) |
| B. m 5 TssG | AIO54744 | 1794749..1822794 | i1 | H. E. Daligault et al. Genome Announc. 2014. 2 (6) |
| B. m 5 TssB | AIO53430 | 1794749..1822794 | i1 | H. E. Daligault et al. Genome Announc. 2014. 2 (6) |
| B. ps 1 TssK | WP_004523698 | 115016..146918 | i3 | M. Kudryashev et al. Cell. 2015. 160 (5), 952-962 |
| B. ps 1 TssG | WP_011205343 | 115016..146918 | i3 | M. Kudryashev et al. Cell. 2015. 160 (5), 952-962 |
| B. ps 1 TssB | WP_004523700 | 115016..146918 | i3 | M. Kudryashev et al. Cell. 2015. 160 (5), 952-962 |
| B. ps 2 TssK | WP_004531523 | 218043..251322 | i3 | M. Kudryashev et al. Cell. 2015. 160 (5), 952-962 |
| B. ps 2 TssG | WP_011205361 | 218043..251322 | i3 | M. Kudryashev et al. Cell. 2015. 160 (5), 952-962 |
| B. ps 2 TssB | WP_004523554 | 218043..251322 | i3 | M. Kudryashev et al. Cell. 2015. 160 (5), 952-962 |
| B. ps 3 TssK | WP_004525552 | 695254..729898 | i3 | M. Kudryashev et al. Cell. 2015. 160 (5), 952-962 |
| B. ps 3 TssG | WP_004190851 | 695254..729898 | i3 | M. Kudryashev et al. Cell. 2015. 160 (5), 952-962 |
| B. ps 3 TssB | WP_004202234 | 695254..729898 | i3 | M. Kudryashev et al. Cell. 2015. 160 (5), 952-962 |
| B. ps 4 TssK | WP_004536933 | 2032502..2067066 | i1 | M. Kudryashev et al. Cell. 2015. 160 (5), 952-962 |
| B. ps 4 TssG | WP_004533373 | 2032502..2067066 | i1 | M. Kudryashev et al. Cell. 2015. 160 (5), 952-962 |
| B. ps 4 TssB | WP_004530571 | 2032502..2067066 | i1 | M. Kudryashev et al. Cell. 2015. 160 (5), 952-962 |
| B. ps 5 TssK | WP_004524805 | 2829708..2858941 | i3 | M. Kudryashev et al. Cell. 2015. 160 (5), 952-962 |
| B. ps 5 TssG | WP_004540225 | 2829708..2858941 | i3 | M. Kudryashev et al. Cell. 2015. 160 (5), 952-962 |
| B. ps 5 TssB | WP_004524810 | 2829708..2858941 | i3 | M. Kudryashev et al. Cell. 2015. 160 (5), 952-962 |
| B. ps 6 TssK | WP_004521949 | 3694948..3719234 | i4b | M. Kudryashev et al. Cell. 2015. 160 (5), 952-962 |
| B. ps 6 TssG | WP_004200010 | 3694948..3719234 | i4b | M. Kudryashev et al. Cell. 2015. 160 (5), 952-962 |
| B. ps 6 TssB | WP_004521952 | 3694948..3719234 | i4b | M. Kudryashev et al. Cell. 2015. 160 (5), 952-962 |
